# *Arabidopsis* PROTEASOME REGULATOR1 is required for auxin-mediated suppression of proteasome activity and regulates auxin signalling

**DOI:** 10.1038/ncomms11388

**Published:** 2016-04-25

**Authors:** Bao-Jun Yang, Xin-Xin Han, Lin-Lin Yin, Mei-Qing Xing, Zhi-Hong Xu, Hong-Wei Xue

**Affiliations:** 1 National Key Laboratory of Plant Molecular Genetics, CAS Center for Excellence in Molecular Plant Sciences, Institute of Plant Physiology and Ecology, Shanghai Institutes for Biological Sciences, Chinese academy of Sciences, Shanghai 200032, People's Republic of China

## Abstract

The plant hormone auxin is perceived by the nuclear F-box protein TIR1 receptor family and regulates gene expression through degradation of Aux/IAA transcriptional repressors. Several studies have revealed the importance of the proteasome in auxin signalling, but details on how the proteolytic machinery is regulated and how this relates to degradation of Aux/IAA proteins remains unclear. Here we show that an *Arabidopsis* homologue of the proteasome inhibitor PI31, which we name PROTEASOME REGULATOR1 (PTRE1), is a positive regulator of the 26S proteasome. Loss-of-function *ptre1* mutants are insensitive to auxin-mediated suppression of proteasome activity, show diminished auxin-induced degradation of Aux/IAA proteins and display auxin-related phenotypes. We found that auxin alters the subcellular localization of PTRE1, suggesting this may be part of the mechanism by which it reduces proteasome activity. Based on these results, we propose that auxin regulates proteasome activity via PTRE1 to fine-tune the homoeostasis of Aux/IAA repressor proteins thus modifying auxin activity.

## Abstract (web summary)

Plant responses to auxin require proteasome-mediated degradation of Aux/IAA transcriptional repressor proteins. Here, Yang *et al*. show that auxin suppresses proteasome activity in a manner dependent on the proteasome regulator PTRE1 and propose a mechanism for fine tuning Aux/IAA homoeostasis.

## Graphical abstract

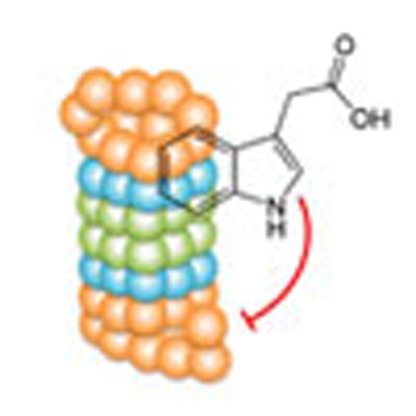

Auxin regulates multiple developmental processes in plants^1^. The F-box protein TRANSPORT INHIBITOR RESPONSE 1 (TIR1) receptor family regulates the transcription of auxin-dependent genes by stimulating degradation of Aux/IAA proteins^2^,^3^, suggesting the proteasome plays a crucial role in regulating Aux/IAA homoeostasis and hence downstream auxin signalling^4^.

The ubiquitin/26S proteasome proteolytic pathway selectively removes regulatory proteins, providing an efficient and rapid strategy to control many cellular processes^5^ and plays critical roles in protein removal in plants^6^,^7^ to regulate various aspects of hormone signalling^8^,^9^, developmental^10^,^11^,^12^,^13^,^14^ and stress responses^15^,^16^. The proteasome is highly conserved and little is known how proteasome activity is regulated in either mammals or plants. The bovine proteasome inhibitor 31 (PI31) (ref. 17) and its homologues in mouse^18^ and humans^19^ diminish the activity of purified 20S proteasome. Interestingly, *Drosophila* PI31 activates 26S proteasome activity and is necessary for sperm differentiation^20^.

Although auxin promotes the interaction of TIR1-Aux/IAAs to target the proteolysis of Aux/IAAs by the 26S proteasome^21^,^22^, whether auxin affects proteasome activity and whether auxin-mediated regulation of proteasome activity regulates plant development remains unclear. Here we report the identification and functional characterization of *Arabidopsis* PROTEASOME REGULATOR1 (PTRE1), which is homologous to human PI31. PTRE1 stimulates 26S proteasome activity and influences auxin-related processes during plant growth and development. We propose that it acts in concert with the TIR1-AFB pathway to buffer the degradation of Aux/IAA proteins and hence modulate the expression of auxin-responsive genes in a precise manner.

## Results

### Identification of PROTEASOME REGULATOR1

**Figure 1:**
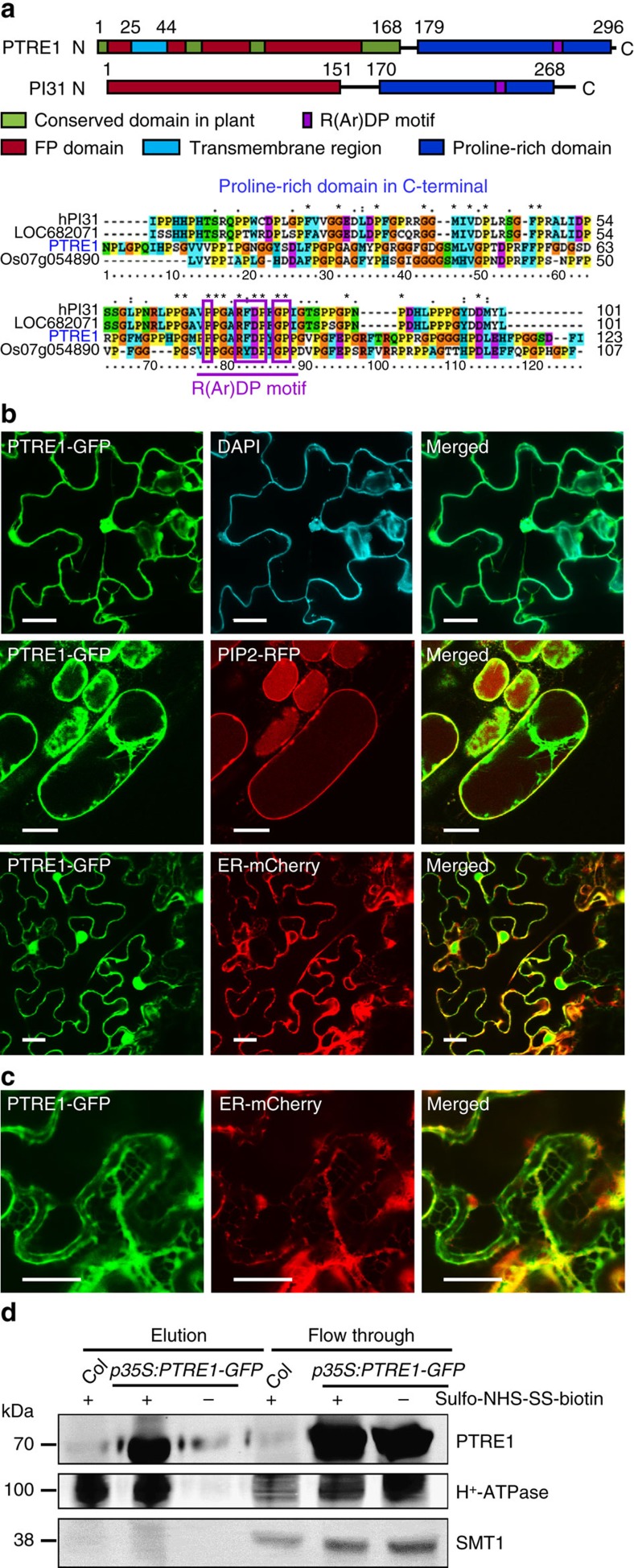
Protein structure and subcellular localization of PTRE1. (**a**) PTRE1 has conserved FP and proline-rich domains similar to human PI31, plant-specific domains and a N-terminal transmembrane region (upper panel). An amino acid alignment of the C-terminal proline-rich domain, which is conserved in the predicted proteasome inhibitor family of eukaryotic species (hPI31, *Homo sapiens*; LOC682071, *Rattus norvegicus*; and Os07g054890, *Oryza sativa*), is shown (bottom panel). The R(Ar)DP motif is indicated by the purple rectangle and the conserved amino acid residues of these four proteins are highlighted with different colours. (**b**) PTRE1 localizes at nucleus (upper; scale bar, 20 μm), plasma membrane (middle; bar, 20 μm) and ER (bottom; bar, 20 μm). Pavement cells of *Arabidopsis* seedlings expressing PTRE1-GFP were stained with DAPI (2 μg ml^−1^) to show the nuclear localization of PTRE1 (upper, DAPI stains nucleus and plant cell walls). Hypocotyl cells of 7-day-old *Arabidopsis* seedlings expressing PTRE1-GFP and plasma membrane aquaporin PIP2-RFP was observed after plasmolysis (middle). Plasmolysis was performed by adding 0.6 M mannitol for 30 min. PTRE1-GFP was transiently expressed in *N. benthamiana* leaves with ER-mCherry (bottom) and observed. (**c**) Enlarged ER localization of PTRE1 by transient expression of PTRE1-GFP or ER-mCherry in *N. benthamiana* leaves. Scale bar, 20 μm. (**d**) Parts of PTRE1 protein were localized and surface-exposed at the plasma membrane. Plant materials were treated with the membrane-impermeable sulpho-NHS-SS-biotin reagent (+). Surface-exposed protein eluted after purification using biotin beads. Plasma membrane protein H^+^-ATPase and ER membrane protein SMT1 were used as control. Equal amounts of samples were subjected to SDS–PAGE and immunoblot analysed using anti-PTRE1 (rabbit), anti-H^+^-ATPase (rabbit) or anti-SMT1 (rabbit) antibodies.

To study the underlying mechanism of how plant proteasome activity is regulated and how auxin-mediated regulation of proteasome activity could potentially regulate plant development, we searched for *Arabidopsis* homologues of the mammalian PI31 protein. We identified a protein encoding a 302 amino acid polypeptide that shares high homology with mammalian PI31, which we designated as PROTEASOME REGULATOR1, (PTRE1). Similar to PI31, PTRE1 has a conserved proline-rich domain at the C-terminus and a highly conserved FP (Fbxo7/PI31) dimerization domain at the N-terminus (Fig. 1a). Interestingly, PTRE1 also contains several other motifs that are highly conserved among plant proteins at the N-terminus that are not present in mammalian PI31 proteins, which may suggest that PTRE1 has distinct functions. Phylogenetic analysis indicated that PTRE1 and its homologues are conserved across different eukaryotes (Supplementary Fig. 1a).

Unlike PI31, prediction of protein secondary structure by SMART reveals the likely presence of a transmembrane region (residues 25–44) and that amino acid residues 45–302 of PTRE1 may be exposed to the outer surface of the plasma membrane (Supplementary Fig. 1b). Subcellular localization analysis revealed that PTRE1 is located at the plasma membrane, the nucleus and the cytoplasm (mainly in endoplasmic reticulum, ER, Fig. 1b,c; Supplementary Fig. 2). Further analysis of surface-exposed protein by using membrane-impermeable sulpho-NHS-SS-biotin showed that PTRE1-GFP and plasma membrane protein H^+^-ATPase were selectively biotinylated, whereas ER protein SMT1 was not (Fig. 1d) indicating the surface accessibility of PTRE1. In contrast, the mammalian PI31 mainly localizes in the cytosol and nucleus^20^, suggesting a possible divergent role of plant proteasome regulators.

### PTRE1 regulates multiple developmental processes

**Figure 2:**
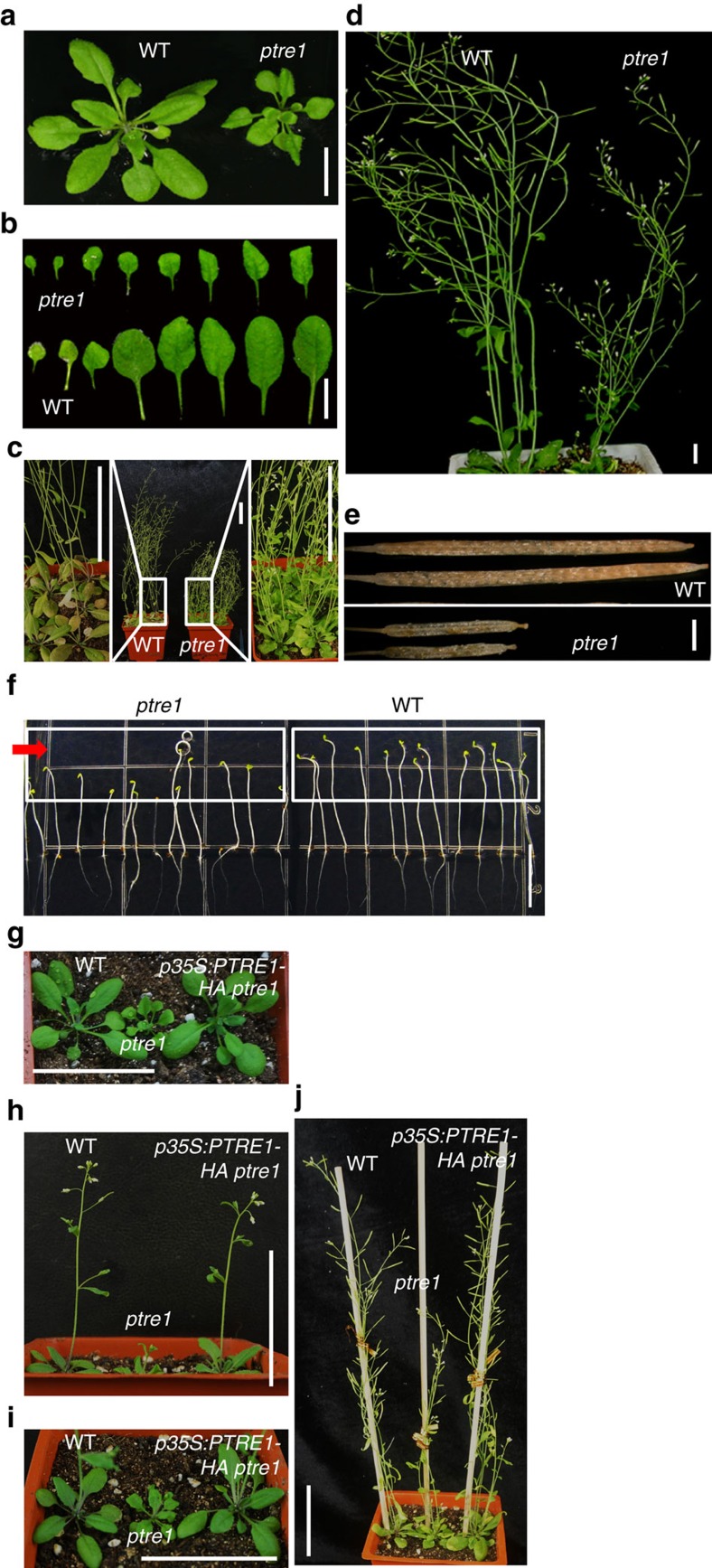
PTRE1 regulates multiple aspects of plant growth. (**a**–**e**) *ptre1* mutant shows multiple auxin-related phenotypes, including suppressed seedling growth (**a**) and leaf development (**b**) reduced apical dominance and increased branches (**c**; 35-day-old plants), delayed flowering and abnormal siliques (**d**; 42-day-old plants). Defective siliques are highlighted (**e**). Scale bar, 1 cm (**a**,**b**,**d**) 1 mm (**e**) or 5 cm (**c**). (**f**) Phenotypic observation of seedling growth under unilateral light showed the suppressed response of *ptre1* mutant. Five-day-old dark-grown wild-type (Col) and *ptre1* seedlings were treated with unilateral light for 12 h and representative images were shown. Arrow indicates the light orientation. Scale bar, 10 mm. (**g**–**j**) Complementary expression of *PTRE1-HA* results in the rescued growth and developmental defects of *ptre1* mutant. Seedlings of wild-type (Col), *ptre1*, *ptre1 p35S:PTRE1-HA* at 2 (**g**) 3 (**h**,**i**) and 5 (**j**) weeks are shown. Scale bar, 5 cm.

To study the physiological function of PTRE1, a putative T-DNA insertion line (SALK_034353) was identified which we named *ptre1*. The T-DNA insertion is located in the first intron of *PTRE1* (Supplementary Fig. 3a,b) and PCR with reverse transcription (RT–PCR) analysis revealed deficient expression of *PTRE1* in *ptre1* mutant (Supplementary Fig. 3c). Moreover, western blot analysis of the homozygous mutant lines revealed the deficiency of PTRE1 protein (Supplementary Fig. 3d), indicating *ptre1* is a knockout mutant. Phenotypic characterization showed that *ptre1* plants were dwarf and displayed developmental defects which are often related to auxin, including small and curved leaves (Fig. 2a,b), altered shoot apical dominance (Fig. 2c), short siliques (Fig. 2d) and arrested embryogenesis (Fig. 2e), which is consistent with the widespread expression of the *PTRE1* gene (Supplementary Fig. 4). In addition, *ptre1* seedlings showed a defective phototropic response (Fig. 2f), a process that involves auxin, suggesting that auxin signalling may be suppressed in *ptre1*. Complementing *PTRE1* expression using *PTRE1* or *PTRE1-GFP* under the control of the 35S promoter rescued the defective growth phenotype of *ptre1* (Fig. 2g–j and Supplementary Fig. 5), confirming the role of PTRE1 in regulating plant growth.

### *ptre1* mutants show altered response to auxin

**Figure 3:**
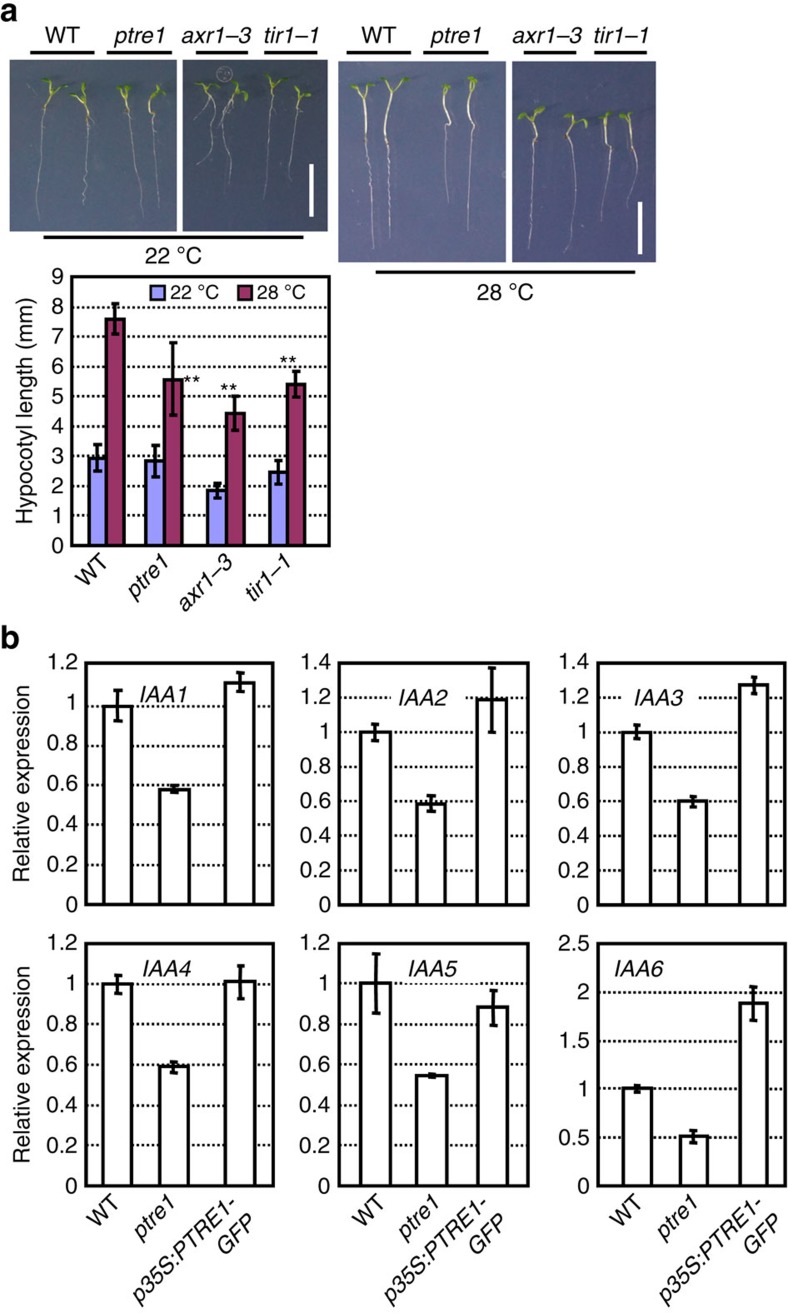
*ptre1* mutants have auxin-related phenotypes. (**a**) *ptre1* shows reduced hypocotyl elongation under high temperature (28 °C). Seedlings were grown at 22 or 28 °C for 6 days after 2 days germination at 22 °C. Seedling growth was observed (upper panel; scale bar, 1 cm) and the hypocotyl length was measured and calculated (bottom panel). Data were presented as means±s.e.m. (*n*>50) and statistical analysis was performed using student's *t-*test (**P<0.01, *ptre1*, *tir1–1* and *axr1–3* compared with wild type). Mutants *axr1–3* and *tir1–1* are shown as positive controls. (**b**) Reduced or enhanced *PTRE1* expression altered the expressions of *IAA* genes (*IAA1–6* are shown). Seven-day-old seedlings of wild-type (Col), *ptre1* and *PTRE1-*overexpressing (*p35S:PTRE1-GFP*) seedlings were analysed and relative expression was calculated using *ACTIN2* as a reference gene. Error bars represent s.d. (*n*=3). The experiments were repeated three times.

Next, we focused on the potential function of PTRE1 in auxin responses. Previous work has shown that auxin promotes hypocotyl elongation at high temperature (28 °C) (ref. 23). We therefore examined hypocotyl elongation in *ptre1* mutant seedlings under high temperature. The results showed that hypocotyl elongation under high temperature (28 °C) was obviously suppressed in *ptre1* (Fig. 3a), although to a lesser extent than in the auxin signalling mutants *tir1–1* and *axr1–3*. In addition, *ptre1* mutants were less sensitive to root growth inhibition by low concentrations of 2,4-D (Supplementary Fig. 6), suggesting a positive role of PTRE1 in auxin signalling. However, we did not detect significant differences in root growth inhibition by NAA or IAA in the *ptre1* mutant, possibly due to the complexity of auxin signalling in this process.

Further examination of *IAA* gene expression in wild-type (Col), *ptre1*, or *PTRE1*-overexpression lines (Supplementary Fig. 7a) showed that compared with wild type, most *IAAs* had reduced expression in *ptre1*, while expression increased in *PTRE1* overexpressers (Fig. 3b and Supplementary Fig. 7b). Auxin rapidly induces the transcription of most *IAA* genes, however, in some cases the induction was significantly suppressed in the *ptre1* mutant, especially after auxin treatments for 3 h (Supplementary Fig. 7c). In other cases, auxin-mediated induction of *IAA* transcription was not altered (or was even stronger) in *ptre1* plants at earlier time points, which may be due to an indirect effect at earlier time points during activation of auxin signalling.

In addition, expression of the *pDR5::GFP* auxin response reporter protein in the *ptre1* mutant was noticeably reduced (Supplementary Fig. 7d,e) despite IAA content being unaltered (Supplementary Fig. 7f). On the basis of these results we suggest that auxin signalling is suppressed in *ptre1* and that PTRE1 has a positive effect on auxin responses. These results indicate that PTRE1 influences the regulation of *IAA* transcription and suggest a possible association of PTRE1 with TIR1/AFBs in mediating auxin signalling.

### Auxin suppression of the 26S proteasome depends of PTRE1

**Figure 4:**
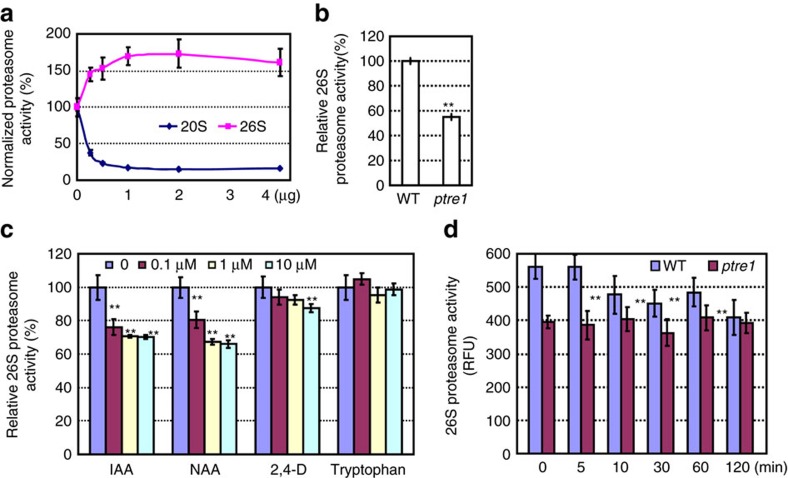
PTRE1 is required for auxin-mediated suppression of proteasome activity. (**a**) Purified PTRE1 protein (recombinantly expressed in *E. coli*) showed inhibitory effects on 20S-proteasome (purified from bovine, Millipore) activity and stimulatory effects on 26S-proteasome (purified from transformed HEK cells, Boston Biochem) activity (proteasome activity without PTRE1 was set as 100% and relative activity was calculated). (**b**) Analysis using total protein extracts of 7-day-old wild-type (Col) and *ptre1* seedlings showed the reduced 26S proteasome activity in *ptre1* mutant. Non-proteasome activity was measured by adding MG132 (Supplementary Fig. 8). The experiments were repeated three times and data is presented as average±s.e.m. Statistical analysis was performed using student's *t*-test (**P<0.01, *n*=9). (**c**–**d**) An assay of 26S proteasome activity showed that auxin suppresses proteasome activity (**c**), which was suppressed in *ptre1* (**d**). Protoplasts of Col were isolated and treated with various concentration of auxin (IAA, NAA or 2,4-D. Tryptophan was used as negative control) for 120 min. In addition, protoplasts of wild type (Col) or *ptre1* were isolated and treated with NAA (1 μM) for different times, then total proteins were extracted and used for measurement of 26S proteasome activity using Suc-LLVY-AMC substrate. Data are presented as average±s.e.m. (*n*=3) and statistical analysis was performed using student's *t*-test (**P<0.01). The experiments were repeated three times.

Biochemical analysis using purified recombinant PTRE1 protein showed that PTRE1 suppresses the 20S but stimulates 26S proteasome activity (Fig. 4a). Consistent with this data, *in vivo* analysis of the proteasome activity in *ptre1* mutants revealed reduced relative 26S proteasome activity and enhanced 20S proteasome activity (Fig. 4b and Supplementary Fig. 8), indicating PTRE1 is indeed an active proteasome regulator in plants. The *ptre1* mutant also displayed a hypersensitive response to MG132 treatment (a proteasome inhibitor; Supplementary Fig. 9).

Considering the auxin-related phenotypes of *ptre1*, we next investigated whether auxin regulates PTRE1 and proteasome activity. Auxin treatment (IAA, NAA or 2,4-D) significantly decreased the 26S proteasome activity in wild type, whereas tryptophan, which has no auxin activity, had no effect (Fig. 4c). Auxin-mediated suppression of 26S proteasome activity was not observed in *ptre1* mutants (Fig. 4d) showing PTRE1 is required for auxin-mediated suppression of proteasome activity. In addition, examination of the 26S proteasome activity at early time points (5, 10 min) showed that the change of proteasome activity is relatively slow (10 min or later) (Fig. 4d). On the basis of these data, we suggest that auxin suppresses proteasome activity via modulation of PTRE1.

### PTRE1 is involved in auxin-mediated Aux/IAA degradation

**Figure 5:**
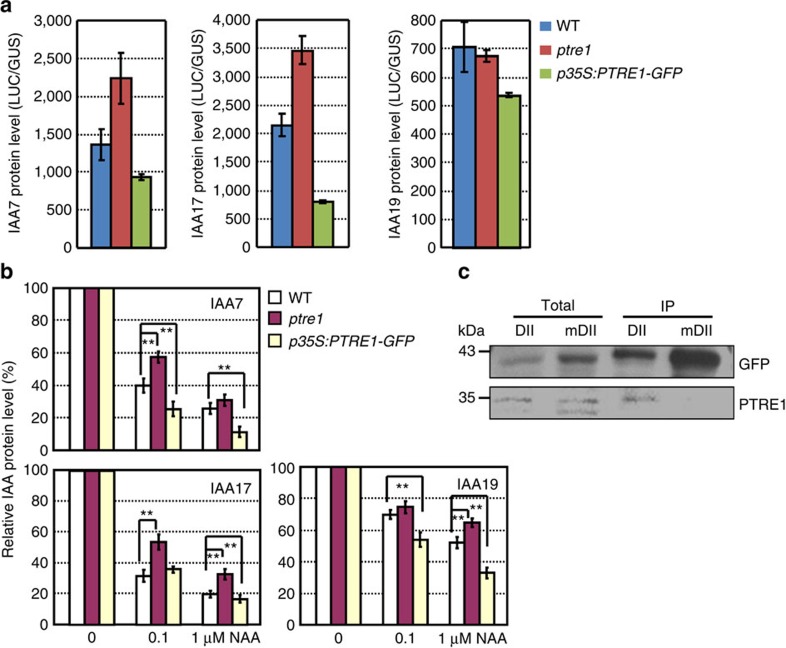
PTRE1 regulates the level of Aux/IAA proteins. (**a**) Increased level of Aux/IAA proteins in *ptre1*. IAA-luciferase fusion proteins (IAA7-luciferase, IAA17-luciferase and IAA19-luciferase) were transiently expressed in protoplasts of wild-type (Col), *ptre1* or PTRE1-overexpressing plants (p35S:PTRE1-GFP) and analysis of the luciferase activity showed that more or less IAA proteins were accumulated in *ptre1* or *p35S:PTRE1-GFP* plants. pUBI10:GUS was co-transformed as an internal control. Data were presented as average±s.d. (*n*=3). The experiments were repeated three times. (**b**) IAA-luciferase fusion proteins (IAA7, IAA17 and IAA19) were transiently expressed in protoplasts of wild-type (Col), *ptre1* or seedlings overexpressing *PTRE1* (p35S:PTRE1-GFP), and calculation of the relative IAA degradation by analysing the luciferase activity showed the decreased or increased degradation rate in *ptre1* or *PTRE1-*overexpressing plants under NAA treatment (0.1 or 1 μM). pUBI10:GUS was co-transformed as an internal control and amount of IAA-luciferase fusion protein without NAA treatment was set as ‘100%'. Data were presented as average±s.d. (*n*=3) and statistical analysis was performed using student's *t*-test (**P<0.01). The experiments were repeated three times. (**c**) Co-immunoprecipitation analysis reveals the association of PTRE1, proteasome and Aux/IAA proteins *in vivo*. Protein extracts from seedlings expressing DII-Venus or mDII-Venus (control) were incubated with anti-GFP antibody conjugated beads and followed by an immunoblot probed with PTRE1 antibody.

To further examine a potential role for PTRE1 in auxin-mediated protein degradation, we analysed the expression of IAA-luciferase fusion proteins (IAA7, IAA17 and IAA19) (Fig. 5a). Protein level of both IAA7 and IAA17 was increased in the *ptre1* mutant and reduced in the *PTRE1*-overexpressing plants, respectively. IAA19 did not over-accumulate in the *ptre1* mutants, but PTRE1-overexpressing lines did exhibit reduced IAA19 levels, which is consistent with PTRE1 functioning in IAA19 degradation.

Further detailed analysis showed altered levels of IAA7, IAA17 and IAA19 in *ptre1* or *PTRE1-*overexpressing plants under auxin treatment (Fig. 5b and Supplementary Fig. 10), confirming that PTRE1 regulates the level of IAA proteins in response to auxin. In addition, co-immunoprecipitation analysis using DII-VENUS^24^, which contains the DII domain found in Aux/IAA proteins, suggests that PTRE1 can associate with IAA proteins *in vivo* (Fig. 5c), further suggesting that PTRE1 can alter Aux/IAA degradation. Notably this interaction was not seen with a mutated version of the DII domain (mDII-VENUS) that cannot be degraded via the proteasome.

### Both PTRE1 and TIR1 regulate auxin signalling

These results indicate that auxin regulates the degradation of IAA proteins not only through the nuclear receptor TIR1 but also through modulation of proteasome activity which requires PTRE1. Further genetic analysis was then performed to investigate the roles of PTRE1 in TIR1-mediated auxin signalling by crossing the *ptre1* and *tir1–1* mutants. Among more than 300 offspring, no homozygous *ptre1 tir1–1* plants were obtained, suggesting that deficiency of both PTRE1 and TIR1 may result in a lethal phenotype.

**Figure 6:**
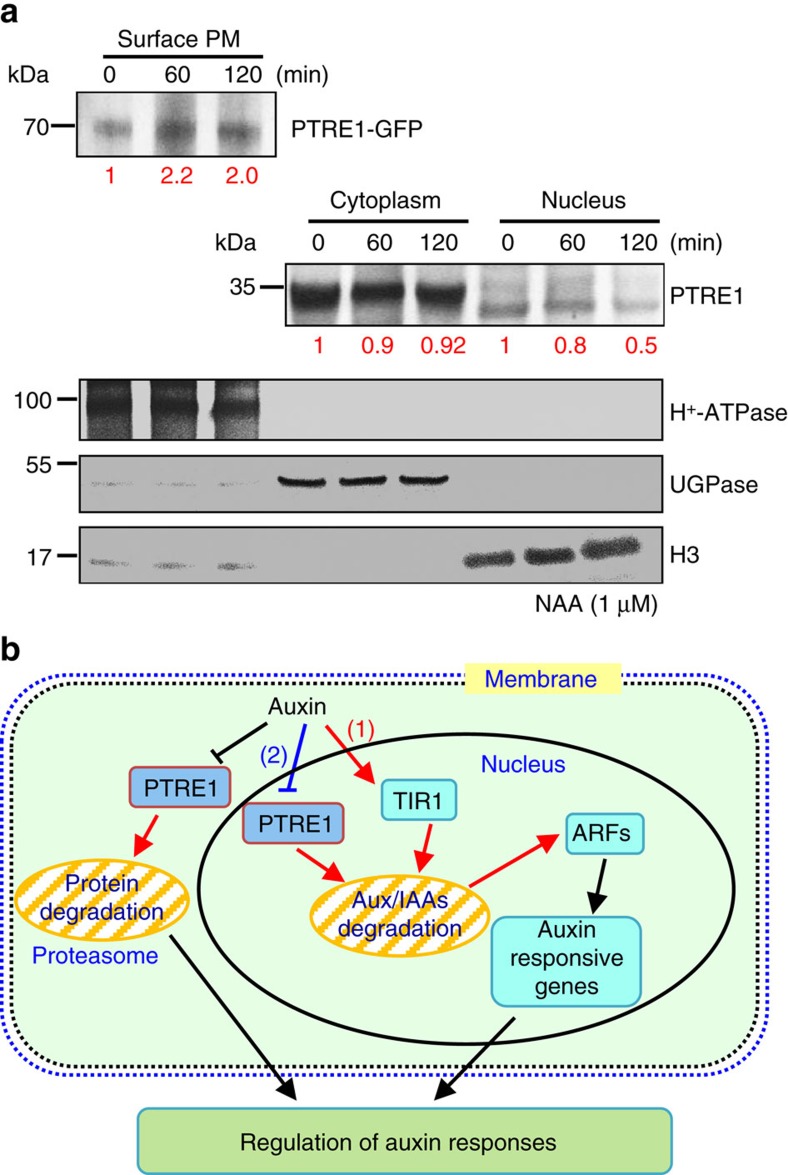
Auxin stimulates PTRE1 accumulation at the plasma membrane. (**a**) Western blot analysis showed that auxin treatment results in relatively more PTRE1 at the plasma membrane, but decreased levels in nucleus and cytoplasm. *Arabidopsis* seedlings expressing PTRE1-GFP were treated with NAA (1 μM, 60 or 120 min) and then surface-exposed membrane proteins were analysed using PTRE1 antibody or plasma membrane marker H^+^-ATPase. Nucleus and cytoplasm fractions were prepared from *Col* seedlings and analysed by western blot using antibody against PTRE1, nuclear marker H3 or cytoplasm marker UGPase. The relative quantities of the proteins were calculated by using image pro plus and indicated. (**b**) A proposed model for how PTRE1 and TIR1 coordinate auxin responses regulating proteasome activity and Aux/IAA protein degradation. Under normal condition (with basal auxin levels), 26S proteasome activity is maintained by appropriate distribution of PTRE1 at the plasma membrane and in intracellular compartments. In response to auxin, auxin rapidly stimulates the association of TIR1 and Aux/IAA proteins resulting in degradation of Aux/IAAs (1). Later, auxin suppresses PTRE1 to inhibit proteasome activity (possibly through stimulating the accumulation of PTRE1 at plasma membrane, resulting in decreased intracellular and nuclear localization) and hence suppresses Aux/IAA protein degradation (2) to coordinate regulation of auxin responses, reflecting a mechanism for fine control of Aux/IAA homeostasis and auxin signalling.

The possible mechanism of how PTRE1 mediates suppression of proteasome activity by auxin was studied. Considering the plasma membrane localization of PTRE1 and that auxin suppresses the endocytosis of membrane proteins^25^, we examined whether auxin modulates the subcellular localization of PTRE1. Western blot analysis showed that NAA treatment results in an increased amount of PTRE1 at the plasma membrane (Fig. 6a) and decreased amounts in internal compartments, especially the nucleus, suggesting that altered localization of PTRE1 in response to auxin may contribute suppression of proteasome activity by auxin.

## Discussion

Auxin promotes the degradation of a large number of Aux/IAA proteins; however, whether auxin affects the proteasome activity and how auxin-mediated regulation of proteasome activity affects plant development is unclear. Our findings suggest auxin can modulate the level of Aux/IAA proteins through regulating 26S proteasome activity. By identifying and functional characterizing PTRE1, which is highly homologous to mammalian PI31, we demonstrated that auxin suppresses proteasome activity. This is dependent on PTRE1, providing new mechanistic insights into auxin signalling and the fine control of Aux/IAA repressor homoeostasis to modulate expression of auxin-responsive genes (Fig. 6b). Compared with the rapid degradation of Aux/IAAs by TIR1, the PTRE1 dependent regulation of the proteasome by auxin occurs relatively slowly (Fig. 4d) and may act to buffer this rapid process, preventing exaggerated degradation of Aux/IAAs. This suggests that PTRE1 and TIR1 activity may be coordinated to mediate auxin responses through regulating Aux/IAA degradation.

The multiple developmental defects including alteration to leaves and shoot apical dominance, arrest of embryogenesis and defective phototropic responses, resemble auxin signalling-deficient mutants^26^ and suggest that PTRE1 may regulate auxin signalling. In addition to auxin, signalling of many other plant hormones including brassinosteroids, abscisic acid, cytokinin, ethylene, gibberellins, jasmonic acid, salicylic acid and strigolactone also involves the ubiqutin-26S proteasome pathway^6^,^27^. Indeed, preliminary analysis revealed increased sensitivity of the *ptre1* mutant to both ABA and brassinolide (BL) (Supplementary Fig. 11), suggesting that PTRE1 may be involved in various hormones signalling pathways by regulating proteasome activity. Interestingly, *ptre1* mutants have different (opposite) responses to auxin and ABA and BL, suggesting that PTRE1 may act as a possible point of cross-talk in coordinating the signalling of various hormones. Considering that auxin-mediated regulation of proteasome activity relies upon PTRE1, how PTRE1 acts in particular signalling pathways (for examples auxin) to regulate proteasome activity at the mechanistic level will be interesting and further investigations on the detailed relevant regulatory mechanism will help to illustrate the distinct regulation of hormone signalling.

Whether PTRE1 functions as a general, rather than a specific, regulator for abnormal and short-lived proteins through stimulating the 26S proteasome is unknown. Auxin's effect on the 26S proteasome activity *in vivo* is relatively modest (∼20–30% decrease). While auxin-mediated repression of the 26S proteasome activity relies upon PTRE1, PTRE1 may presumably have many auxin-independent functions. In addition, deficiency of PTRE1 results in increased or decreased activity of 20S or 26S proteasome *in vivo*, and compared with the disruption of the *PI31* gene in *Drosophila* which results in blocked embryonic development^20^, *Arabidopsis ptre1* mutant could survive but shows severe developmental defects during the entire life cycle, suggesting a divergent role of proteasome activity regulation in plants. This is consistent with the presence of ∼700 F-box proteins in plants^28^, which is much more than that in animals. Up to now, it is still not conclusive whether mammalian PI31 proteins stimulate 26S proteasome *in vivo*^29^. In mammalian cells there is no change in the overall cellular proteasome content or function when PI31 levels are either increased or decreased by RNAi, and thus the cellular roles and mechanisms of PI31 in regulating proteasome function remain unclear and require further definition. The *ptre1* mutant shows severe developmental defects, suggesting that PTRE1-regualted proteasome activity is required for normal plant growth and development, providing an effective system to study the cellular roles and functional mechanisms of PI31 homologues.

The proteasome is localized in both cytoplasm and nucleus^30^, and the altered PTRE1 localization in response to auxin suggests that altered subcellular localization of PTRE1 may contribute to the regulation of proteasome activity by auxin. In addition, other regulatory mechanisms may be involved in the regulation of PTRE1. Recent studies showed that ADP-ribosylation of DmPI31 results in stimulated proteasome activity^31^, thus whether auxin suppresses proteasome activity through regulating PTRE1 post-translational modification requires further investigation. In addition, interaction with other proteins may be involved in the regulation of PTRE1 activity as well. The C-terminal proline-enriched domain of PTRE1 is conserved in different (putative) proteasome regulators among various species (Fig. 1a). It has been shown that the proline residues occur in numerous structural motifs and many of which are involved in specific protein–protein interactions^32^, and it is possible that PTRE1 may interact with other regulatory proteins to be regulated or to participate in various processes. In addition, the CDP (conserved domain in plant) motifs^33^, which are highly conserved in plant proteins, are especially prominent in PTRE1 (Fig. 1a), suggesting that PTRE1 may be regulated by a different mechanism than in animals.

Although proteasome-mediated protein degradation is crucial in various signalling pathways and in developmental control, little is known how proteasome activity is regulated. It has been reported that PI31 may interact with subunits of 20S proteasome to inhibit its activity by competitive binding. In addition, *in vitro* analysis showed that PI31 stimulates the proteasome activity much strongly than PTRE1 (the 26S proteasome activity was enhanced up to threefold with DmPI31 recombinant protein, but was increased less than twofold with recombinant PTRE1 protein in our assay), which may because of the presence of HbYX motif in PI31 (ref. 20) (which does not exist in PTRE1 protein), suggesting the differences between PTRE1 and mammalian homologues. *Drosophila* DmPI31 was shown to be regulated by the F-box protein Nutcracker^20^. Further studies of how PTRE1 affects proteasome activity will facilitate the understanding of the regulatory mechanism of proteasome activity.

In addition, our preliminary analysis showed that poly-ubiquitinated proteins are accumulated in *ptre1* (Supplementary Fig. 12), confirming that PTRE1 is necessary for maintaining the normal 26S proteasome activity; however, the protein level of RGA1 (ref. 34), a poly-ubiquitinated protein degraded by 26S proteasome, did not accumulate in *ptre1*, suggesting that PTRE1 may be selective in regulating protein degradation. Further studies of how PTRE1 affects proteasome activity and how PTRE1 is regulated by other factors will help to illustrate the mechanism how specific proteins may be degraded through proteasome selectivity/specificity regulation, which will facilitate the understanding of the regulatory mechanism of proteasome activity in plants and expand the knowledge of distinct protein/pathway regulations at post-translational level.

## Methods

### Materials and growth conditions

*Arabidopsis thaliana* ecotype Columbia (Col) was used in all experiments. All seeds were germinated on MS (Murashige and Skoog, Duchefa) medium after three days at 4 °C. Seedlings and plants were grown in a phytotron at 22 °C with a 16 h light/8 h dark photoperiod. Root growth measurements were performed using 7-day-old seedlings grown on media containing different concentrations of NAA, IAA or 2,4-D.

The *ptre1* mutant (SALK 034353) was obtained from the SALK Institute^35^ and was genotyped using primers LB, RP and LP. Homozygous *ptre1* line was firstly identified by RT–PCR using primers (PTRE1–5′ and PTRE1–3′) and the candidate lines were further confirmed by western blot analysis using antibody against PTRE1 (rabbit). Primer sequences are listed in Supplementary Table 1.

For auxin treatment, seedlings was transferred to liquid 1/2 MS medium containing NAA (1 μM) for different times before RNA extraction. For MG132 treatment, 3-day-old normal growth seedlings on MS media were transferred to medium containing MG132 for another 3 days. For treatment with BL, seedlings were grown vertically on MS medium containing different concentrations of BL (0.1, 1, 10 and 100 nM) in darkness for 7 days and then the hypocotyls lengths were measured. For ABA treatment, plant grown at different concentrations of ABA (0.2, 0.5 and 1 μM) after 10 days was observed.

### Plasmid construction and *A. thaliana* transformation

For promoter-reporter gene (GUS) fusion, a 2 kb promoter region of *PTRE1* was amplified by PCR (primers PTRE1-p5′ and PTRE1-p3′) using *Arabidopsis* genomic DNA as template.

The PTRE1 cDNA was amplified by PCR with primers PTRE1–5′ and PTRE1–3′ using total cDNA of *Arabidopsis* seedlings as template and subcloned into pCAMBIA1302 to generate the p35S:PTRE1-GFP construct. For transient expression in *Nicotiana benthamiana* leaves, PTRE1 cDNA was subcloned into pENTR/D-TOPO (Invitrogen) and then LR reactions with pGWB5, pGWB14 or pGWB654 to obtain the p35S:PTRE1-GFP, p35S:PTRE1-HA and p35S:PTRE1-RFP constructs.

Transformation of Col or *ptre1* plants was performed by the floral dipping procedure. GUS activity was detected according to the previous description^36^.

To generate the IAA-Luciferase constructs (IAA7, IAA17 and IAA19), the GFP coding sequence of pA7 vector (kindly provided by Dr K. Czempinski, University of Potsdam, Germany) was replaced with that of luciferase (amplified by primers pA7-LUC-P1 and pA7-LUC-P2) and then cDNA of IAAs (amplified by primers pA7-IAA7/17/19-P1 and pA7-IAA7/17/19-P2) were subcloned in the resultant vector, respectively. To generate pUBI10:GUS construct, the CaMV35S promoter of pA7 was replaced with UBI10 promoter (amplified by primers UBI10-P1 and UBI10-P2 using *Arabidopsis* genomic DNA as template).

Primers are listed in Supplementary Table 1.

### Quantitative real-time RT–PCR analysis

Total RNAs were extracted from Col or *ptre1* seedlings using TRIzolR reagent (Invitrogen), incubated with DNAase (TaKaRa) and reverse transcribed (Toyobo). Transcription of *IAAs* and *ACTIN2* genes was analysed using the SYBR Green qPCR kit (Toyobo) with a RotorGene 3,000 system (Corbett). The primers were previously described^37^ or listed in Supplementary Table 1. Relative expression of examined genes was calculated by setting the gene expression level of wild type as ‘1' and presented as average±s.d. from three independent biological replicates.

### Measurement of the free IAA content

Free IAA content was measured by using a Thermo TSQ Quantum Ultra LC-MS-MS system according to previous description^38^. Briefly, shoots (∼150 mg) of Col or *ptre1* were frozen in liquid N2 and ground into a fine powder with a mortar and pestle. Following the addition of 600 μl of methanol, homogenates were mixed and kept at 4 °C overnight, then centrifuged at 4,800*g* for 10 min. The supernatant was transferred to a new glass tube and the residue was re-extracted with 200 μl of methanol. Three millilitre ddH_2_O was added to the combined extracts, which were then passed through the Waters Sep-pak C_18_ cartridge. The cartridge was washed with 200 μl 20% methanol and 250 μl 30% methanol to discard the eluent. Finally, the extract was collected by eluting the cartridge with 300 μl methanol.

Solutions with IAA at concentrations of 10,100 and 1,000 ng ml^−1^ were used as standards. Samples were analysed by a Thermo TSQ Quantum Ultra LC-MS-MS system and 10 μl of the sample was injected onto a Hypersil Gold column (150 × 2.1, 3 μm). The mobile phase comprised solvent A (0.1% formic acid) and solvent B (methanol) was used in a gradient model (time/concentration of A/concentration of B, min/%/%, for 0/90/10; 1/90/10; 10/10/90; 15/10/90; 16/90/10; and 28/90/10). Other parameters were set as follows: electrospray voltage, 4,800 V; atomization flow, 30 ml min^−1^; auxiliary flow, 2 ml min^−1^; capillary transfer temperature, 380 °C; lens compensation voltage, 77 V; in-source collision flow, 0 ml min^−1^; molecular ions *m/z*, IAA; collision energy, 15 eV; and signal collection, 15–19 min.

### Subcellular localization and co-localization studies

PTRE1-GFP, PTRE1-RFP and ER-mCherry^39^ fusion proteins were transiently expressed in *N. benthamiana* leaves^40^. The infiltrated leaves were harvested 2 days after infiltration and observed using a Olympus confocal microscope (Olympus, FV10i). The leaves transiently expressed PTRE1-RFP was counterstained with dihydrochloride (DAPI, 2 μg ml^−1^, Invitrogen) to visualize the nucleus. Stably transformed *A. thaliana* plants expressing PTRE1-GFP was also used to observe the localization of PTRE1.

Images were captured with the following excitation (Ex) and emission (Em) wavelengths (Ex/Em): GFP 488 nm/501–528 nm; mCherry/RFP 543 nm/620–630 nm; and DAPI 405 nm/437–476 nm. Stably transformed *A. thaliana* plants were observed after plasmolysis.

### Plasmolysis

Plasmolysis was performed by treating seedlings with protoplasting solution for 30 min before imaging. A fresh protoplasting solution was prepared as follow: 0.3% Macerozyme (Yakult), 0.6 M D-mannitol, 20 mM MES monohydrate and 20 mM KCl. The solution was first warmed up for 10 min at 55 °C, then cooled down at room temperature. 10 mM CaCl_2_ was added before use.

### Proteasome activity assay

The 20S Proteasome Activity Assay kit (Millpore) was used to measure the *in vitro* or *in vivo* activity of 20S proteasome. Briefly, the *in vivo* 20S proteasome activity was measured as follows: 7-day-old *Arabidopsis* seedlings of *Col* and *ptre1* were ground in protein-extraction buffer (50 mM Tris, pH 7.5, 150 mM NaCl, 1% Triton-100 and 20% glycerol) and cell debris were removed by centrifugation at ∼13,000*g* (4 °C). After determining the protein concentration by BCA (bicinchoninic acid, Pierce, BCA Protein Assay Kit), protein (100 μg) from crude extracts of each sample was diluted with 10 × assay buffer (250 mM HEPES, pH 7.5, 2 mM DTT, 5 mM EDTA, 0.5% NP-40 and 0.01% SDS, supplied in the 20S Proteasome Activity Assay kit) to a final volume of 1 ml for measuring the 20S proteasome activity (assayed in quadruplicate). Hydrolysis of fluorogenic proteasome substrates SUC-LLVY-AMC (Millpore) was monitored at excitation 380 nm and emission 460 nm. *In vitro* activity of 26S Proteasome assay was carried out as described^20^. Briefly, 0.05 μg of 26S proteasome (purified from transformed HEK cells, Boston Biochem) was used per 100 μl reaction buffer (50 mM Tris, 5% glycerol, 10 mM MgCl_2_ and 100 μM ATP). The fluorogenic peptide substrate SUC-LLVY-AMC (Millpore) was used at a final concentration of 100 μM in reaction buffer.

*In vivo* 26S proteasome activity assay was performed according to previous description^41^. In detail, 7-day-old *Arabidopsis* seedlings or well-expanded leaf protoplasts from 3–4-week-old plants were treated with 1 μM NAA for different times. Collected samples were ground in homogenization buffer (50 mM Tris, pH 7.5, 150 mM NaCl, 5 mM ATP, 1% Triton-100 and 20% glycerol) and cell debris were removed by centrifugation at 10,000*g* (4 °C). Protein concentration was determined by BCA assay. To measure the 26S proteasome activity, protein (100 μg) from crude extracts of each sample was diluted with buffer I (50 mM Tris, pH 7.4, 2 mM DTT, 5 mM MgCl_2_ and 2 mM ATP) to a final volume of 1 ml (assayed in quadruplicate). Samples were incubated with the proteasome substrate LLVY or pre-incubated with MG132 (25 μM, for measuring non-proteasome background) for 30 min at room temperature before adding LLVY, and then the proteasome activity was measured. The exact 26S or 20S proteasome activity was obtained by subtracting the activity of non-proteasome (with MG132) from the overall measured values.

Suc-LLVY-AMC was added to a final concentration of 80 μM and proteolytic activities were monitored per 100 μl at fluorescence plate reader (380 nm excitation and 460 nm emission filters) after 2 h incubation at 37 °C. All reactions were conducted in a 96-well plate and read by a Varioskan Flash micro-plate reader (Thermo Scientific).

### Isolation and analysis of cell surface proteins

Isolation and immunoblot analysis of cell surface proteins were performed using cell surface protein isolation kit (Pierce) according to previous description^42^. In detail, seedlings were grown in 1/2 MS medium for 9 days and aerial parts (∼0.5 g) were harvested and rinsed in PBS solution (20 ml). After incubation in PBS (10 ml, with 0.4 mM sulpho-NHS-SS-biotin, Pierce) at room temperature for 1 h, unreacted sulpho-NHS-SS-biotin was quenched with Quenching solution (500 μl, Pierce) followed by twice 20 min washes in TBS. Aerial parts were then collected and ground into fine powder in liquid nitrogen and then suspended in 5 ml of lysis buffer (20 mM HEPES, 100 mM NaCl, 5 mM MgCl_2_, Cocktail). Cell lysate was spun at 2,000*g* for 10 min (4 °C) to pellet the cell debris and supernatant was spun at 100,000*g* for 1 h (4 °C) to pellet the cell membranes. Membranes were then solubilized in 500 μl of membrane lysis buffer (Pierce) with cocktail, and 100 μl were removed for analysis of the total membrane proteins. Biotinylated, solubilized membrane proteins were bound to 250 μl of Neutravidin-agarose beads (Pierce) for 1 h and washed five times with 500 μl wash buffer (Pierce). Neutravidin-bound, biotinylated proteins were eluted with 400 μl of SDS–polyacrylamide gel electrophoresis (SDS–PAGE) sample buffer (62.5 mM Tris-HCl at pH6.8, 1% SDS and 10% glycerol) containing 50 mM dithiothreitol. Total membrane, biotinylated and flow through proteins were analysed by SDS–PAGE and western blot.

Replication of the results in Fig. 6a are shown in Supplementary Fig. 14 and replicate results of Supplementary Figs 7a,e and 12 are shown in Supplementary Fig. 16. Full uncropped images of all gels and blots are shown in Supplementary Figs 13 and 15.

Antibodies used in this study include anti-GFP (cat-sc-9996, 1:2,000, Santa or cat-598, 1:2,000, MBL); anti-H^+^-ATPase (cat-AS07260, 1:1,000, Agrisera), anti-SMT1 (cat-AS07 266, 1:1,000, Agrisera), anti-PTRE1 (1:1,000).

### Recombinant expression of PTRE1 and antibody generation

*PTRE*1 cDNA was amplified using primers PTRE128a5′ and PTRE128a3′. The amplified fragment was cloned into the pET-28a(+) vector (Novagen) to generate PTRE1–6 × His-tag construct. After confirmation by sequencing, the construct was transformed into *Escherichia coli* Rosetta cells, and expression of the fusion protein was induced by adding isopropyl-β-D-thiogalactoside (final concentration 1 mM) at 37 °C. The cells were lysed by sonication in lysis buffer (50 mM NaH_2_PO_4_, 300 mM NaCl and 10 mM imidazole, pH 8.0) and PTRE1-His protein was purified using Ni-NTA Agarose (Qiagen) under native conditions. His-tag antibody (cat-M089, 1: 5,000, MBL) was used to confirm the expression of recombinant PTRE1 in *E. coli*.

For PTRE1 antibody generation, the purified PTRE1-His (pET-28a-PTRE1) protein was used to immunize rabbits more than three times, and the serum-antibody titre was detected by enzyme-linked immunosorbent assay. The antiserum was collected and isolated to obtain PTRE1 antibody (Abmart). All research involving animals complied with protocols approved by the Biomedical Research Ethics Committee, Shanghai Institutes for Biological Sciences.

### Isolation of nucleus and cytoplasm fractions

The nucleus fraction of 10-day-old Col seedlings were extracted using Sigma CellLytic PN extraction kit. The soluble cytoplasmic protein was isolated as below: seedlings were ground to fine powder in liquid N2. The powder was further ground in cold grinding buffer (20 mM Tris-HCl (pH 8.8), 150 mM NaCl, 1 mM EDTA, 20% glycerol and cocktail). The sample was spun at 6,000*g* for 30 min at 4 °C and the resultant supernatant was further spun at 100,000*g* for 1 h at 4 °C to pellet the total membrane fraction. The resultant supernatant was referred as soluble cytoplasm fraction. Antibodies used include anti-H3 (cat-AS10710, 1:1,000, Agrisera), anti-UGPase (cat-AS142813, 1:1,000, Agrisera), anti-H^+^-ATPase (cat-AS07260, 1:1,000, Agrisera) and anti-PTRE1 (1:1,000).

### Protoplast transfection and fluorometric LUC and GUS assays

Protoplast transfection and activity assays of IAA (IAA7, IAA17 and IAA19)-LUC fusion proteins in Col and *ptre1* were performed according to the previous description^43^,^44^. Briefly, leaves from Col, *ptre1* or *PTRE1*-overexpressing (p35S:PTRE1-GFP) plants (3–4 weeks) were harvested to isolate protoplasts and 300 μl of protoplasts suspension (∼2 × 10^5^ protoplasts) was transfected with constructs p35S::IAA-Luc (30 μg) and pUBI10::GUS (6 μg, for experimental normalization). Transfected protoplasts were incubated at 22 °C for 12 h in the presence of NAA or IAA. After collection and lysis, the LUC activity was measured using 20 μl lysate and 100 μl LUC assay reagent (Promega) with luminometer (Varioskan Flash micro-plate reader, Thermo Scientific). The GUS fluorescence activity was measured using 10 μl lysate and 100 μl MUG substrate mix for 2 h at 37 °C with a fluorometer (365/455, Varioskan Flash micro-plate reader, Thermo Scientific). Each experiment was repeated three times and at least three measurements were performed for each sample.

### Ubiquitin conjugate levels assay

To analyse the ubiquitin conjugate levels, total protein was extracted from 7-day-old Col (one group was treated with 10 μM MG132 for 2 h), *ptre1* and *PTRE1-*overexpressing (p35S:PTRE1-GFP) seedlings, then subjected to SDS–PAGE and immunoblot analysis with antibodies. Antibodies used include anti-Ub (cat-sc-8017, 1:1,000, Santa); anti-RGA1 (cat-AS111630, 1:1,000, Agrisera); anti-PBA1 (cat- BML-PW0430, 1:1,000, Enzo); anti-RPN12A (cat-BML-PW0440, 1:1,000, Enzo); anti-RPN6 (cat-BML-PW8370, 1:1,000, Enzo); anti-ACTIN (cat-M20009, 1:5,000, Abmart); and anti-PTRE1 (1:1,000).

### Co-IP analyses

Immunoprecipitation (IP) of DII-VENUS was performed according to the previous description^45^. Briefly, plants expressing DII-VENUS or mDII-VENUS were ground to powder in liquid nitrogen and solubilized with extraction/washing buffer (50 mM Tris-HCl, pH 7.4, 100 mM NaCl, 10 mM MgCl_2_, 1 mM EDTA, 10% glycerol, 1 mM DTT, 1 mM PMSF and complete protease inhibitor) with GFP beads for 1.5 h. The beads were washed and resuspended with loading buffer for SDS–PAGE and western bolting analysis. Antibody against GFP (cat-sc-9996, 1:100, Santa) or PTRE1 (1:1,000) was used for IP or western blot analysis, respectively.

## Additional information

**How to cite this article:** Yang, B.-J. *et al*. *Arabidopsis* PROTEASOME REGULATOR1 is required for auxin-mediated suppression of proteasome activity and regulates auxin signalling. *Nat. Commun.* 7:11388 doi: 10.1038/ncomms11388 (2016).

## Supplementary Material

Supplementary Information Supplementary Figures 1-16 and Supplementary Table 1

## References

[b1] Weijers D. & Friml J. SnapShot: auxin signalling and transport. Cell 136, 1172 (2009). 19303857 10.1016/j.cell.2009.03.009

[b2] Kepinski S. & Leyser O. The *Arabidopsis* F-box protein TIR1 is an auxin receptor. Nature 435, 446–451 (2005). 15917798 10.1038/nature03542

[b3] Dharmasiri N., Dharmasiri S. & Estelle M. The F-box protein TIR1 is an auxin receptor. Nature 435, 441–445 (2005). 15917797 10.1038/nature03543

[b4] Eckardt N. A. Auxin and the power of the proteasome in plants. Plant Cell 13, 2161–2163 (2001). 11595793 10.1105/tpc.131010 PMC526025

[b5] Mogk A., Schmidt R. & Bukau B. The N-end rule pathway for regulated proteolysis: prokaryotic and eukaryotic strategies. Trends Cell Biol. 17, 165–172 (2007). 17306546 10.1016/j.tcb.2007.02.001

[b6] Vierstra R. D. The ubiquitin-26S proteasome system at the nexus of plant biology. Nat. Rev. Mol. Cell. Biol. 10, 385–397 (2009). 19424292 10.1038/nrm2688

[b7] Smalle J. & Vierstra R. D. The ubiquitin 26S proteasome proteolytic pathway. Annu. Rev. Plant Biol. 55, 555–590 (2004). 15377232 10.1146/annurev.arplant.55.031903.141801

[b8] Smalle J. . The pleiotropic role of the 26S proteasome subunit RPN10 in *Arabidopsis* growth and development supports a substrate-specific function in abscisic acid signalling. Plant Cell 15, 965–980 (2003). 12671091 10.1105/tpc.009217 PMC152342

[b9] Smalle J. . Cytokinin growth responses in *Arabidopsis* involve the 26S proteasome subunit RPN12. Plant Cell 14, 17–32 (2002). 11826296 10.1105/tpc.010381 PMC150548

[b10] Ueda M. . The HALTED ROOT gene encoding the 26S proteasome subunit RPT2a is essential for the maintenance of *Arabidopsis* meristems. Development 131, 2101–2111 (2004). 15073153 10.1242/dev.01096

[b11] Cho Y. H., Yoo S. D. & Sheen J. Regulatory functions of nuclear hexokinase1 complex in glucose signalling. Cell 127, 579–589 (2006). 17081979 10.1016/j.cell.2006.09.028

[b12] Jin H. L., Li S. T. & Villegas A. Down-regulation of the 26S proteasome subunit RPN9 inhibits viral systemic transport and alters plant vascular development. Plant Physiol. 142, 651–661 (2006). 16905670 10.1104/pp.106.083519 PMC1586039

[b13] Huang W. H. . The proteolytic function of the *Arabidopsis* 26S proteasome is required for specifying leaf adaxial identity. Plant Cell 18, 2479–2492 (2006). 17028202 10.1105/tpc.106.045013 PMC1626615

[b14] Brukhin V., Gheyselinck J., Gagliardini V., Genschik P. & Grossniklaus U. The RPN1 subunit of the 26S proteasome in *Arabidopsis* is essential for embryogenesis. Plant Cell 17, 2723–2737 (2005). 16169895 10.1105/tpc.105.034975 PMC1242268

[b15] Wang S. H., Kurepa J. & Smalle J. A. The *Arabidopsis* 26S proteasome subunit RPN1a is required for optimal plant growth and stress responses. Plant Cell Physiol. 50, 1721–1725 (2009). 19605416 10.1093/pcp/pcp105

[b16] Sung D. Y., Kim T. H., Komives E. A., Mendoza-Cozatl D. G. & Schroeder J. I. ARS5 is a component of the 26S proteasome complex, and negatively regulates thiol biosynthesis and arsenic tolerance in *Arabidopsis*. Plant J. 59, 802–812 (2009). 19453443 10.1111/j.1365-313X.2009.03914.x PMC2830867

[b17] Ma C. P., Slaughter C. A. & Demartino G. N. Identification, purification, and characterization of a protein activator (Pa28) of the 20S proteasome (Macropain). J. Biol. Chem. 267, 10515–10523 (1992). 1587832

[b18] Zaiss D. M. W., Standera S., Holzhutter H., Kloetzel P. M. & Sijts A. J. A. M. The proteasome inhibitor PI31 competes with PA28 for binding to 20S proteasomes. FEBS Lett. 457, 333–338 (1999). 10471803 10.1016/s0014-5793(99)01072-8

[b19] McCutchen-Maloney S. L. . cDNA cloning, expression, and functional characterization of PI31, a proline-rich inhibitor of the proteasome. J. Biol. Chem. 275, 18557–18565 (2000). 10764772 10.1074/jbc.M001697200

[b20] Bader M. . A conserved F-box regulatory complex controls proteasome activity in *Drosophila*. Cell 145, 371–382 (2011). 21529711 10.1016/j.cell.2011.03.021 PMC3108249

[b21] Tan X. . Mechanism of auxin perception by the TIR1 ubiquitin ligase. Nature 446, 640–645 (2007). 17410169 10.1038/nature05731

[b22] Gray W. M., Kepinski S., Rouse D., Leyser O. & Estelle M. Auxin regulates SCF^TIR1^-dependent degradation of AUX/IAA proteins. Nature 414, 271–276 (2001). 11713520 10.1038/35104500

[b23] Gray W. M., Ostin A., Sandberg G., Romano C. P. & Estelle M. High temperature promotes auxin-mediated hypocotyl elongation in *Arabidopsis*. Proc. Natl Acad. Sci. USA 95, 7197–7202 (1998). 9618562 10.1073/pnas.95.12.7197 PMC22781

[b24] Brunoud G. . A novel sensor to map auxin response and distribution at high spatio-temporal resolution. Nature 482, 103–132 (2012). 22246322 10.1038/nature10791

[b25] Paciorek T. . Auxin inhibits endocytosis and promotes its own efflux from cells. Nature 435, 1251–1256 (2005). 15988527 10.1038/nature03633

[b26] Mockaitis K. & Estelle M. Auxin receptors and plant development: a new signalling paradigm. Annu. Rev. Cell Dev. Biol. 24, 55–80 (2008). 18631113 10.1146/annurev.cellbio.23.090506.123214

[b27] Chapman E. J. & Estelle M. Mechanism of auxin-regulated gene expression in plants. Annu. Rev. Genet. 43, 265–285 (2009). 19686081 10.1146/annurev-genet-102108-134148

[b28] Xu G. X., Ma H., Nei M. & Kong H. Z. Evolution of F-box genes in plants: different modes of sequence divergence and their relationships with functional diversification. Proc. Natl Acad. Sci. USA 106, 835–840 (2009). 19126682 10.1073/pnas.0812043106 PMC2630105

[b29] Li X. H., Thompson D., Kumar B. & DeMartino G. N. Molecular and cellular roles of PI31 (PSMF1) protein in regulation of proteasome function. J. Biol. Chem. 289, 17392–17405 (2014). 24770418 10.1074/jbc.M114.561183 PMC4067172

[b30] Kurepa J. & Smalle J. A. Structure, function and regulation of plant proteasomes. Biochimie 90, 324–335 (2008). 17825468 10.1016/j.biochi.2007.07.019

[b31] Cho-Park P. F. & Steller H. Proteasome regulation by ADP-ribosylation. Cell 153, 614–627 (2013). 23622245 10.1016/j.cell.2013.03.040 PMC3676968

[b32] Tang X. . Suprafacial orientation of the SCFcdc4 dimer accommodates multiple geometries for substrate ubiquitination. Cell 129, 1165–1176 (2007). 17574027 10.1016/j.cell.2007.04.042

[b33] Marchler-Bauer A. . CDD: a conserved domain database for protein classification. Nucleic Acids Res. 33, D192–D196 (2005). 15608175 10.1093/nar/gki069 PMC540023

[b34] Dill A., Thomas S. G., Hu J. H., Steber C. M. & Sun T. P. The *Arabidopsis* F-box protein SLEEPY1 targets gibberellin signalling repressors for gibberellin-induced degradation. Plant Cell 16, 1392–1405 (2004). 15155881 10.1105/tpc.020958 PMC490034

[b35] Alonso J. M. . Genome-wide insertional mutagenesis of *Arabidopsis thaliana*. Science 301, 653–657 (2003). 12893945 10.1126/science.1086391

[b36] Jefferson R. A., Kavanagh T. A. & Bevan M. W. Gus fusions-beta-glucuronidase as a sensitive and versatile gene fusion marker in higher plants. EMBO J. 6, 3901–3907 (1987). 3327686 10.1002/j.1460-2075.1987.tb02730.x PMC553867

[b37] Braun N. . Conditional repression of AUXIN BINDING PROTEIN1 reveals that it coordinates cell division and cell expansion during postembryonic shoot development in *Arabidopsis* and tobacco. Plant Cell 20, 2746–2762 (2008). 18952781 10.1105/tpc.108.059048 PMC2590743

[b38] Zhang S. C. . Perturbation of auxin homoeostasis caused by mitochondrial FtSH4 gene-mediated peroxidase accumulation regulates *Arabidopsis* architecture. Mol. Plant 7, 856–873 (2014). 24482432 10.1093/mp/ssu006

[b39] Nelson B. K., Cai X. & Nebenfuhr A. A multicolored set of *in vivo* organelle markers for co-localization studies in *Arabidopsis* and other plants. Plant J. 51, 1126–1136 (2007). 17666025 10.1111/j.1365-313X.2007.03212.x

[b40] Lin D. S. . A ROP GTPase-dependent auxin signalling pathway regulates the subcellular distribution of PIN2 in *Arabidopsis* roots. Curr. Biol. 22, 1319–1325 (2012). 22683260 10.1016/j.cub.2012.05.019 PMC3407329

[b41] Pan J., Zhang Q., Wang Y. A. & You M. 26S Proteasome activity is down-regulated in lung cancer stem-like cells propagated *in vitro*. PLoS ONE 5, e13298 (2010). 20949018 10.1371/journal.pone.0013298 PMC2952619

[b42] Park S., Szumlanski A. L., Gu F., Guo F. & Nielsen E. A role for CSLD3 during cell-wall synthesis in apical plasma membranes of tip-growing root-hair cells. Nat. Cell Biol. 13, 973–980 (2011). 21765420 10.1038/ncb2294

[b43] Tao L. Z., Cheung A. Y. & Wu H. M. Plant Rac-like GTPases are activated by auxin and mediate auxin-responsive gene expression. Plant Cell 14, 2745–2760 (2002). 12417698 10.1105/tpc.006320 PMC152724

[b44] Yoo S. D., Cho Y. H. & Sheen J. *Arabidopsis* mesophyll protoplasts: a versatile cell system for transient gene expression analysis. Nat. Protoc. 2, 1565–1572 (2007). 17585298 10.1038/nprot.2007.199

[b45] Feng S. . The COP9 signalosome interacts physically with SCFCOI1 and modulates jasmonate responses. Plant Cell 15, 1083–1094 (2003). 12724535 10.1105/tpc.010207 PMC153718

