# Supplementary material for: Arabidopsis PROTEASOME REGULATOR1 is required for auxin-mediated suppression of proteasome activity and regulates auxin signalling
Source: Nat Commun. 2016 Apr 25;7:11388. doi: 10.1038/ncomms11388 (PMC4848511; doi:10.1038/ncomms11388)
Supplement: Supplementary Information — Supplementary Figures 1-16 and Supplementary Table 1 [file ncomms11388-s1.pdf]

(a)

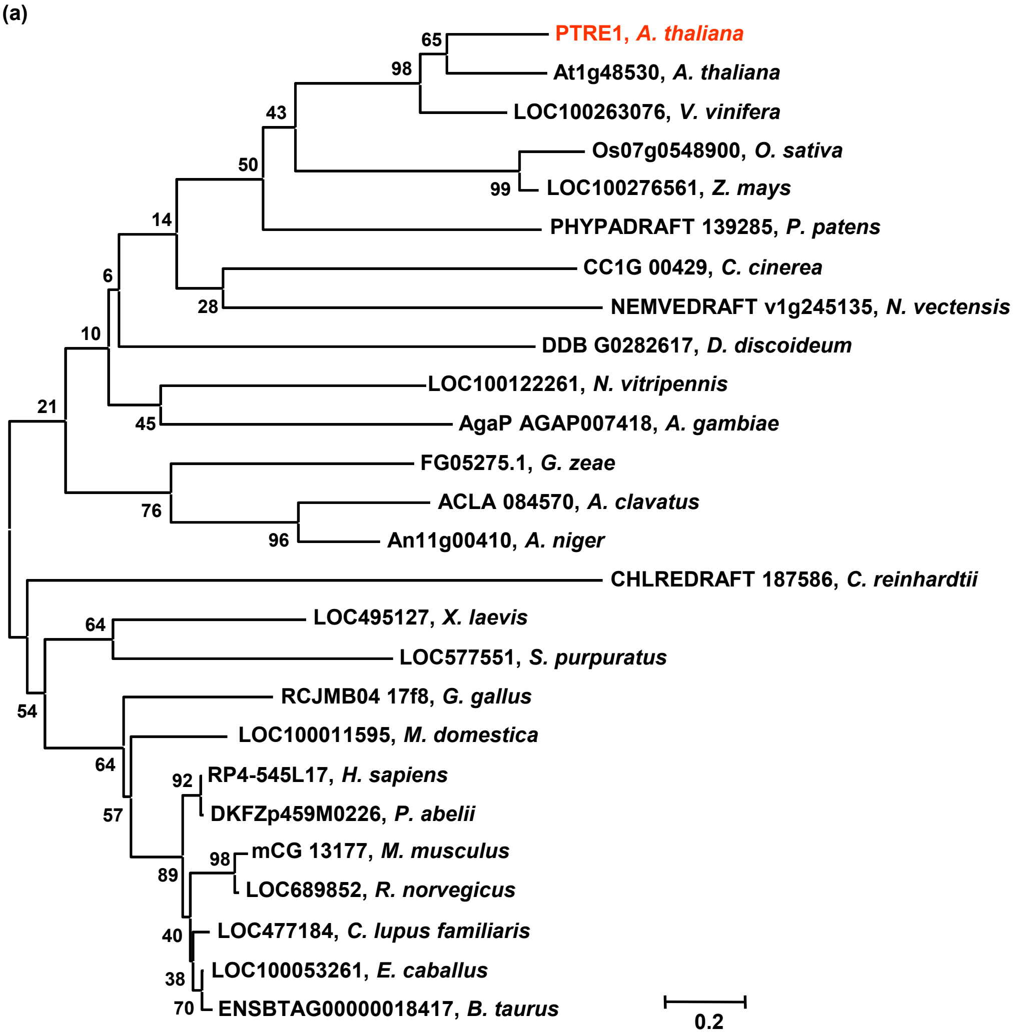

(b)

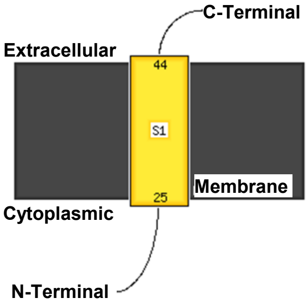

Supplementary Figure 1. Phylogenetic analysis of (putative) proteasome inhibitors of different species.

(a) The phylogenetic tree was produced by Molecular Evolutionary Genetics Analysis 4.1 (MEGA4.1, downloaded from http://www.megasoftware.net/). Support was assessed from 1000 bootstrap replicates and the scale bar corresponds to 0.2 estimated amino acid substitutions per site.

(b) Analysis by secondary protein structure predication (SMART) reveals PTRE1 a membrane protein and amino acid residues 25-44 of PTRE1 is a transmembrane region, amino acid residues 45-302 of PTRE1 localizes to outer surface of the plasma membrane. The topological prediction displays that C-terminal is exposed to the outer surface of the plasma membrane.

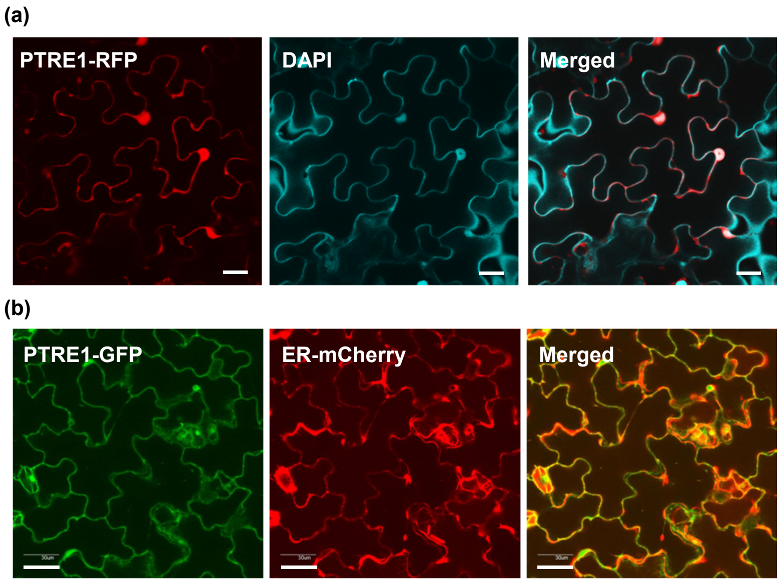

Supplementary Figure 2. PTRE1 is localized at nucleus and ER.
(a) PTRE1-RFP was transiently expressed in *N. benthamiana* leaves and observation revealed the localization of PTRE1 in nucleus. Bar=20 μm.
(b) PTRE1-GFP was transiently expressed in *Arabidopsis* leaves with ER-mCherry and observation revealed the ER localization of PTRE1. Bar=30 μm.

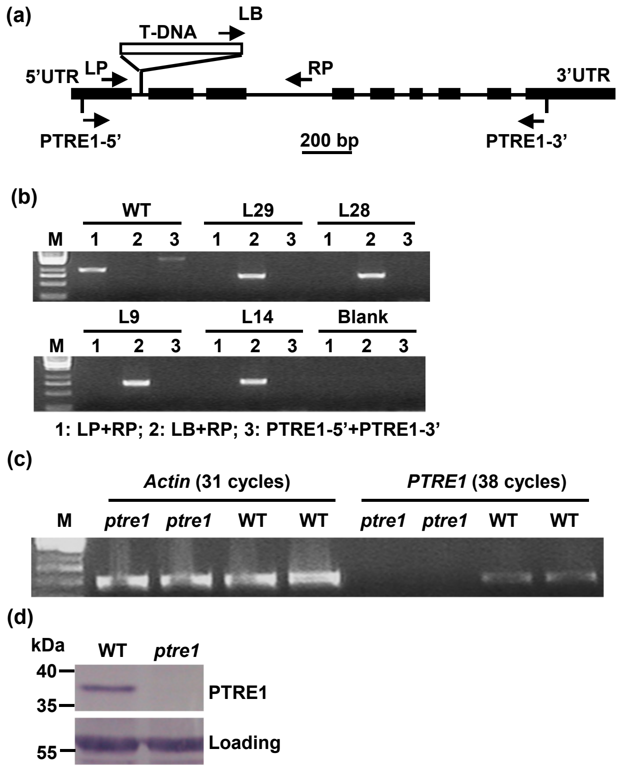

Supplementary Figure 3. Confirmation of *ptre1* mutant.

(a) Structure of the *PTRE1* gene (nine exons) and position of T-DNA insertion of the *ptre1* mutant.

(b) Genomic DNAs from independent plants (L29, 28, L9, L14) of *ptre1* mutant were used as template for PCR amplification. Homozygous lines have a single amplified fragment of 700 bp.

(c) Analysis by RT-PCR revealed the deficiency of *PTRE1* transcripts in *ptre1* plants. Primers PTRE1-5' and PTRE1-3' are used and positions of primers are indicated in (a).

(d) Detection of the PTRE1 protein amount by western blot analysis using specific antibody against PTRE1 confirmed *ptre1* a knock-out mutant. The unspecific band was used as loading control.

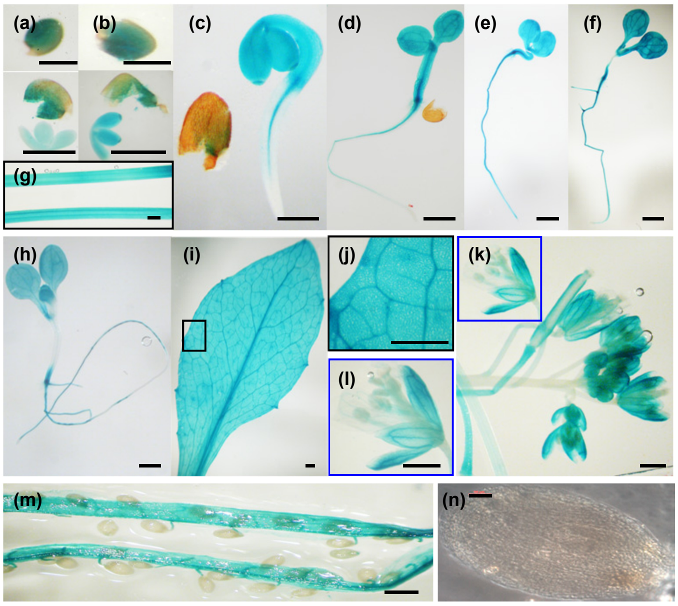

Supplementary Figure 4. Expression pattern analysis of *PTRE1*. Promoter-reporter gene fusion studies reveal the expression of *PTRE1* in germinating seeds (immersed in water for 4 h, a), young seedlings (b-g), stem (h), leaf (i, the squared region is enlarged in j), flower (k, the squared region is enlarged in l), and silique (m). There is no detectable *PTRE1* expression in developing seeds (n, late heart stage of embryo). Bars = 0.5 mm (a-m) or 50 μm (n). Activity of β-glucuronidase (GUS) driven by 2.0-kb *PTRE*1 promoter was examined. Three independent transgenic lines were analyzed.

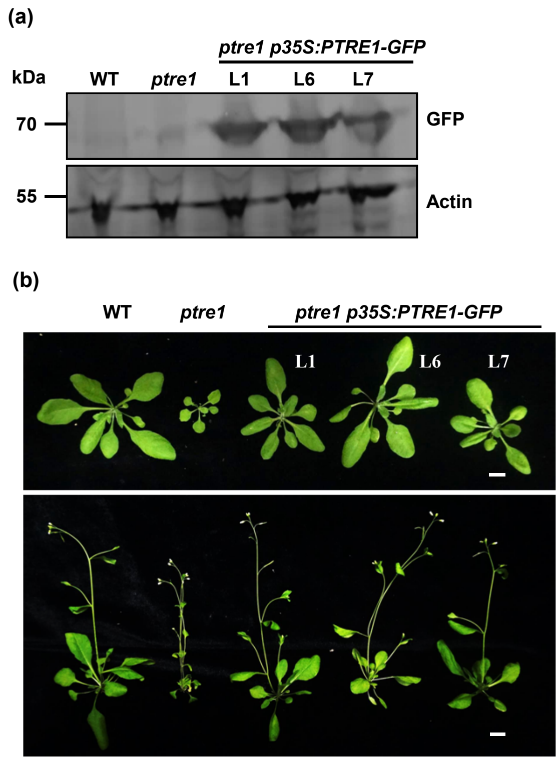

Supplementary Figure 5. Expression of *PTRE1-GFP* results in the rescued growth and developmental defects of *ptre1* mutant.

(a) Western blot analysis using antibody against GFP confirmed the PTRE1-GFP expression in *ptre1 p35S:PTRE1-GFP* plants. Equal amounts of protein were subjected to SDS-PAGE and immunoblot analysis was performed using antibodies again ACTIN or GFP. ACTIN was used as loading control (bottom).

(b) Seedlings of wild type (Col), *ptre1*, *ptre1 p35S:PTRE1-GFP* (lines L1, L6, L7) at two (upper) or four (bottom) weeks were shown. Bar=1 cm.

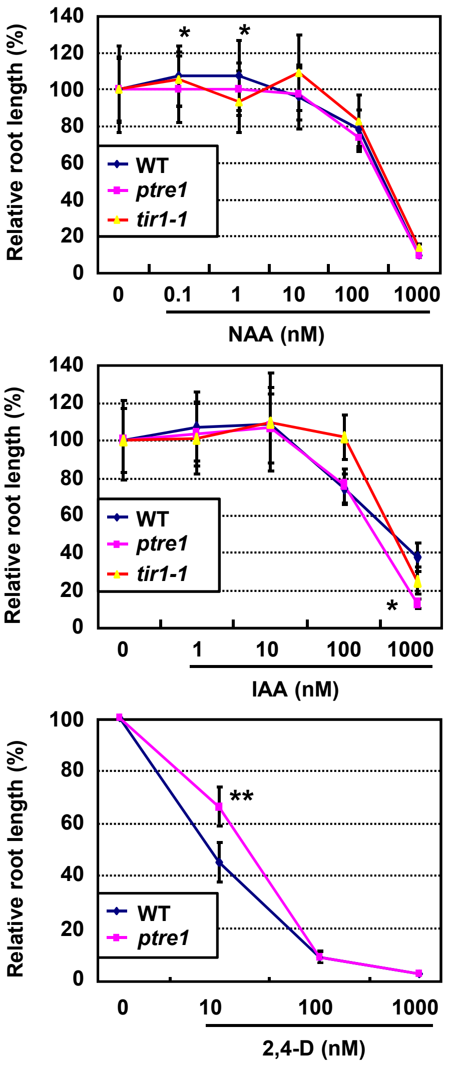

Supplementary Figure 6. *ptre1* mutants are less sensitive to auxin-mediated root growth suppression. Root growth at different concentrations of NAA, IAA or 2,4-D was measured and relative length was calculated. Data were presented as means±SE (n>50) and statistical analysis by student's *t*-test revealed the significant difference (*, *P*<0.05, **, *P*<0.01, compared to wild type). *tir1-1* mutant is used as a positive control.

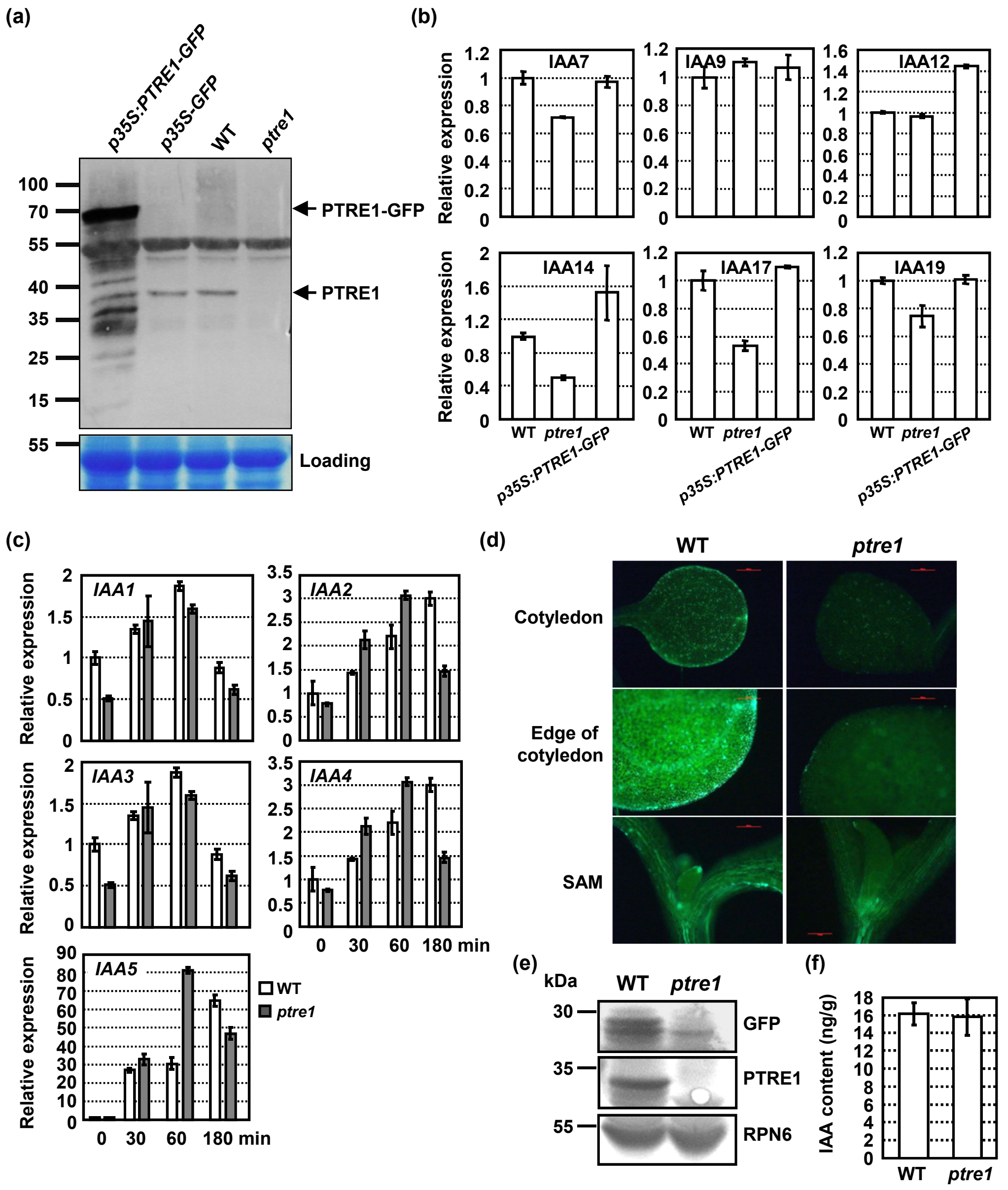

Supplementary Figure 7. *ptre1* presents suppressed auxin signaling.

(a) Western blotting analysis confirmed the expression of PTRE1-GFP in wild-type (Col). Protein levels of PTRE1-GFP in Col, *ptre1* and p35S:PTRE1-GFP were detected by immunoblot analysis using antibodies again PTRE1. Protein extracted from one-week-old plants were subjected to SDS-PAGE and Coomassie staining showed the equal amounts of the proteins.

(b) Reduced or enhanced *PTRE1* expression altered the expressions of *IAA* genes. Seven-day-old seedlings of wild type (Col), *ptre1* and *PTRE1*-overexpressing (p35S:PTRE1-GFP) lines were analyzed and the relative expression of *IAA* genes was calculated with *ACTIN2*. Error bars represent SD (n=3). The experiments were repeated for three times.

(c) *ptre1* showed suppressed auxin-induced expression of *IAA* genes (*IAA1-5* were analyzed), indicating a positive effect of PTRE1 on auxin signaling and IAA regulation. Seven-day-old seedlings of wild type (Col) and *ptre1* were collected and treated in liquid MS containing 1 μM NAA for 0, 30, 60 or 180 min. Data were normalized with *ACTIN2* and relative expression was calculated by setting the expression at time 0 as "1". Error bars represent SD (n=3). The experiments were repeated for three times.

(d) Observation of the pDR5:GFP expression patterns reveals the suppressed auxin signaling of *ptre1* mutant. Four-day-old seedlings of wild type (Col) and *ptre1* mutant expressing pDR5:GFP were observed. Similar results were obtained in at least three independent *ptre1* pDR5:GFP cross offsprings and representative results were shown. Bar=200 μm.

(e) Decreased protein level of GFP in *ptre1* mutant expressing pDR5:GFP. Three-week-old wild type (Col) and *ptre1* plants expressing pDR5:GFP were used for analysis. RPN6 was used as loading control. Equal amounts of samples were subjected to SDS-PAGE and immunoblot analysis was performed using anti-PTRE1 (rabbit), anti-GFP or anti-RPN6 antibodies.

(f) Unaltered auxin content of *ptre1*. ~150 mg shoots of 7-day-old wild type (Col) and *ptre1* seedlings were used to measure the free IAA content. Data were presented as means±SD (n=3). The experiments were biologically repeated for four times.

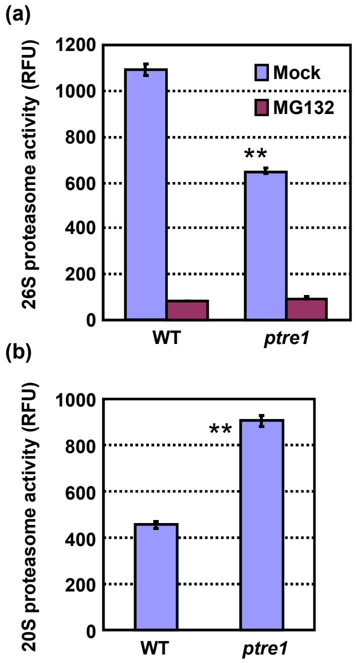

Supplementary Figure 8. Altered proteasome activity in *ptre1* seedlings. Proteasome inhibitor MG132 was applied to exclude the non-proteasome background and analysis confirmed the decreased 26S proteasome **(a)** or increased 20S proteasome **(b)** activity of *ptre1*. Proteasome of 7-day-old wild type (Col) and *ptre1* seedlings was extracted and incubated with proteasome substrate LLVY or pre-incubated with MG132 (25 μM) for 30 min at room temperature before adding LLVY. When measuring the activity of 20S proteasome, assay buffer (Millpore) containing Sodium Dodecyl Sulfate (SDS, 0.001%) was added for activation of the 20S Proteasome. The experiments were repeated three times and data is presented as average±SE. Statistical analysis by student's *t*-test revealed the significant difference (**, P<0.01).

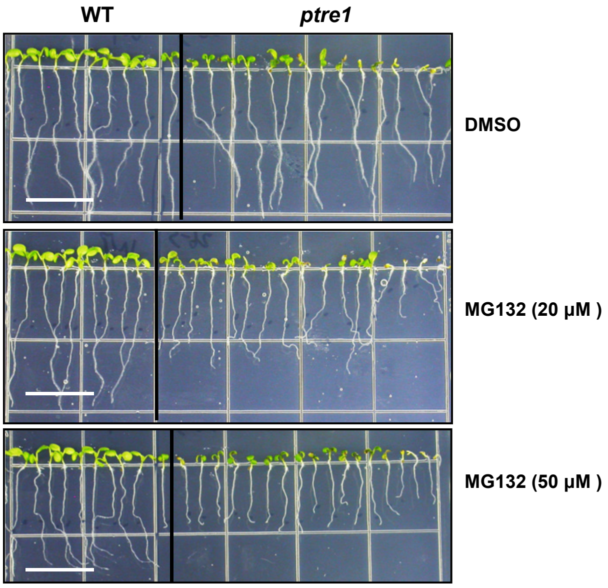

Supplementary Figure 9. Observation of the seedling root growth reveals the hypersensitive response of *ptre1* to proteasome inhibitor MG132. Wild type (Col) and *ptre1* seedlings were grown in MS medium for 3 days and transferred to medium supplemented with MG132 (20 or 50 µM) for 3 days and observed. Bar=1.35 cm.

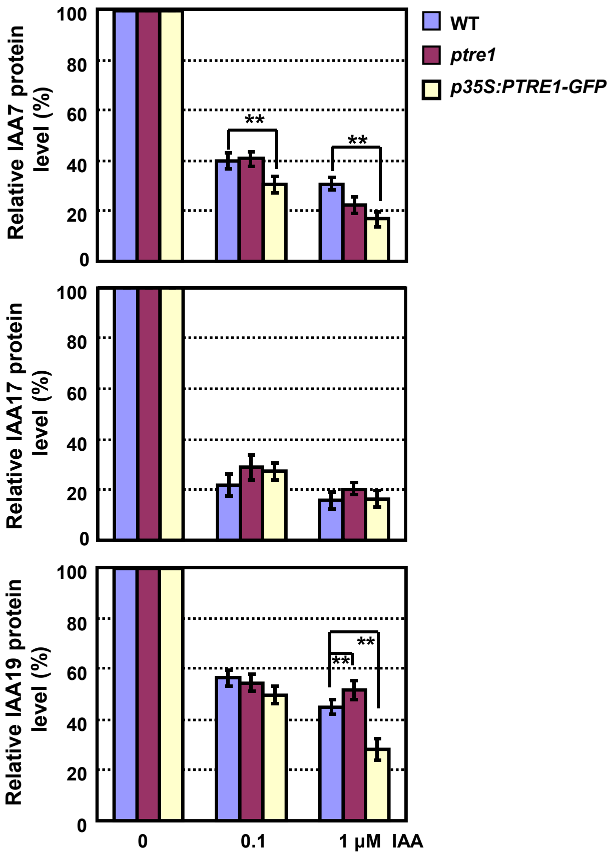

Supplementary Figure 10. Degradation of IAA proteins in *ptre1* under IAA treatment. IAA-luciferase fusion proteins (IAA7-luciferase, IAA17-luciferase, IAA19-luciferase) were transiently expressed in protoplasts of wild type (Col), *ptre1* or *PTRE1*-overexpressing (p35S:PTRE1-GFP) plants and the relative accumulation of IAA proteins under IAA treatment (0.1 or 1 μM), by analyzing the luciferase activity, was shown. pUBI10:GUS was co-transformed as an internal control. Amount of IAA-luciferase fusion protein without IAA treatment was set as “100%” and relative protein level was calculated. Data were presented as average±SD (n=3) and statistical analysis was performed by student’s *t*-test (**, P<0.01). The experiments were repeated for three times.

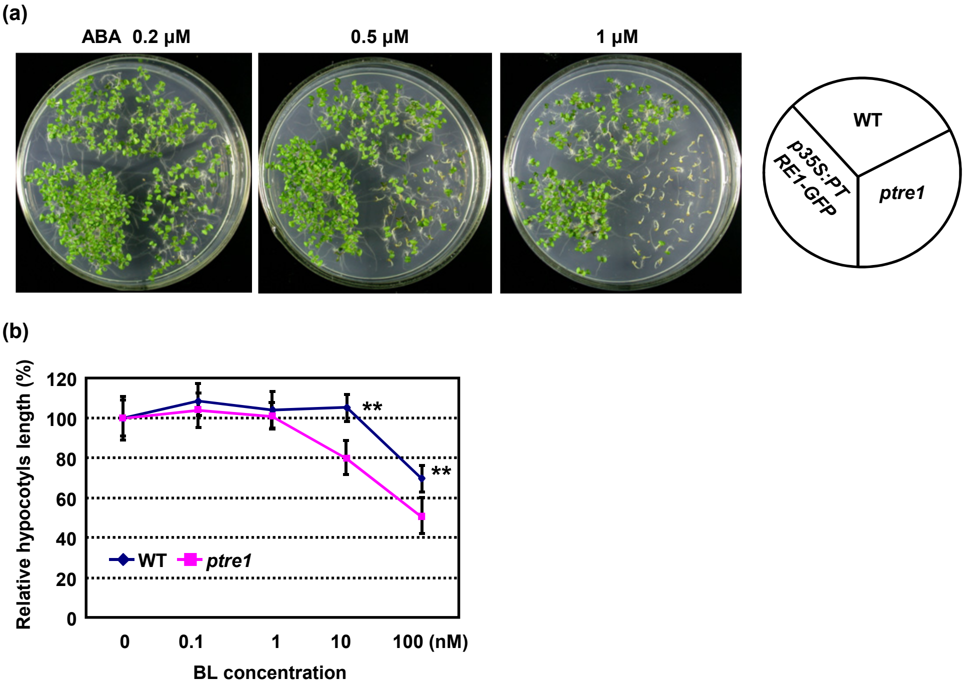

Supplementary Figure 11. *ptre1* is hypersensitive to BL and ABA.

(a) Observation showed that *ptre1* is hypersensitive to ABA treatment. Seedlings were grown under different concentrations of ABA for 10 days.

(b) *ptre1* is more sensitive to BL treatment by analyzing the hypocotyl elongation. Seedlings were grown vertically on MS medium containing different concentrations of BL in darkness or light for 7 days and hypocotyl length was measured. Relative hypocotyl length was calculated by setting the length absence of BL as "100%". Data were presented as means ± SD (n>30, **, P<0.01).

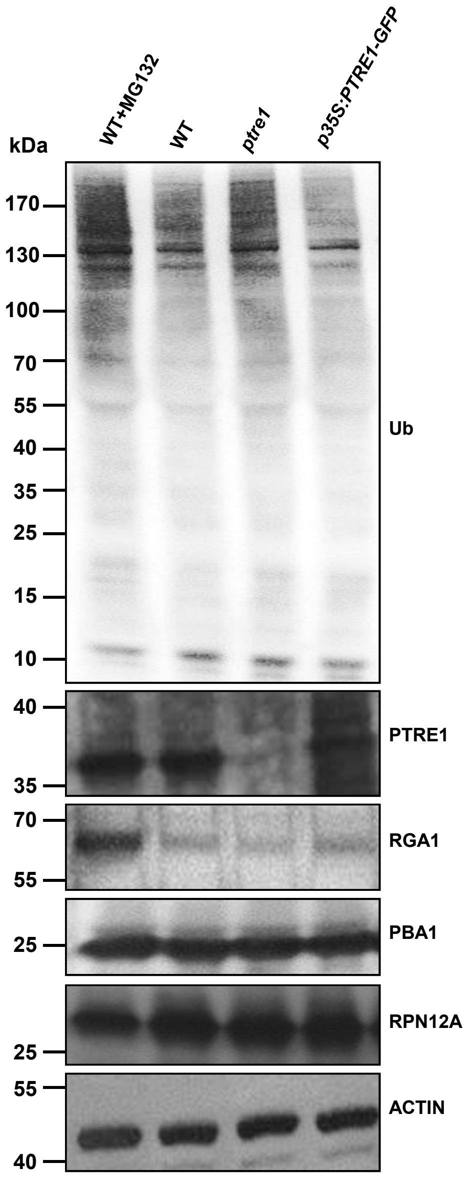

Supplementary Figure 12. Ubiquitin conjugate levels of RGA1 were not accumulated in *ptre1*. The protein levels of RGA1 and proteasome subunits PBA1 and RPN12A in *ptre1* were detected. Equal amounts of protein extracted from one-week-old plants were subjected to SDS-PAGE and immunoblot analysis was performed using antibodies again ACTIN, Ubiquitin (Ub), PTRE1, PBA1, RPN12A, or RGA1. ACTIN was used as loading control.

Full uncropped images of Fig. 1d

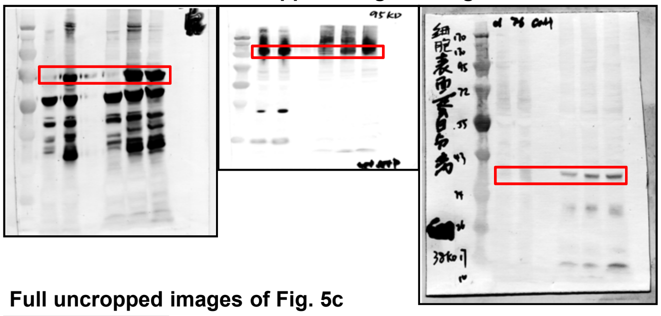

Full uncropped images of Fig. 5c

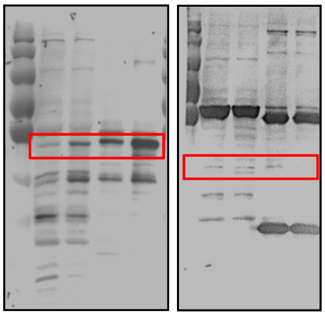

Full uncropped images of Fig. 6a

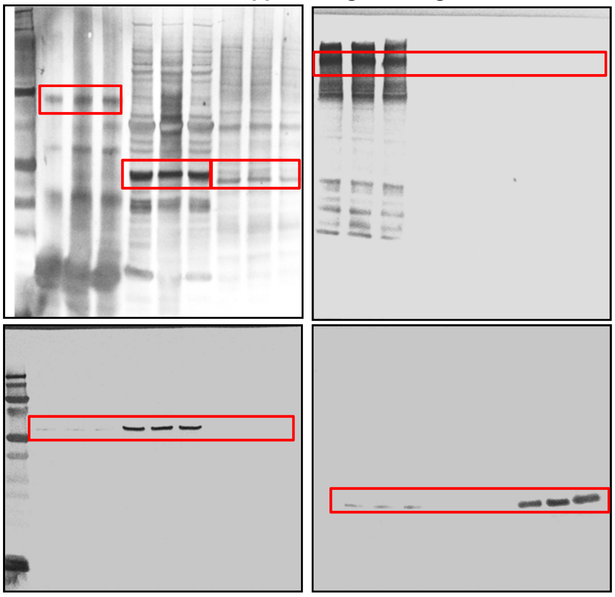

Supplementary Figure 13. Full uncropped images of gels and blots. The cropped bands shown in figures **1d, 5c and 6a** are indicated by red frames.

Replicate blots of Fig. 6a

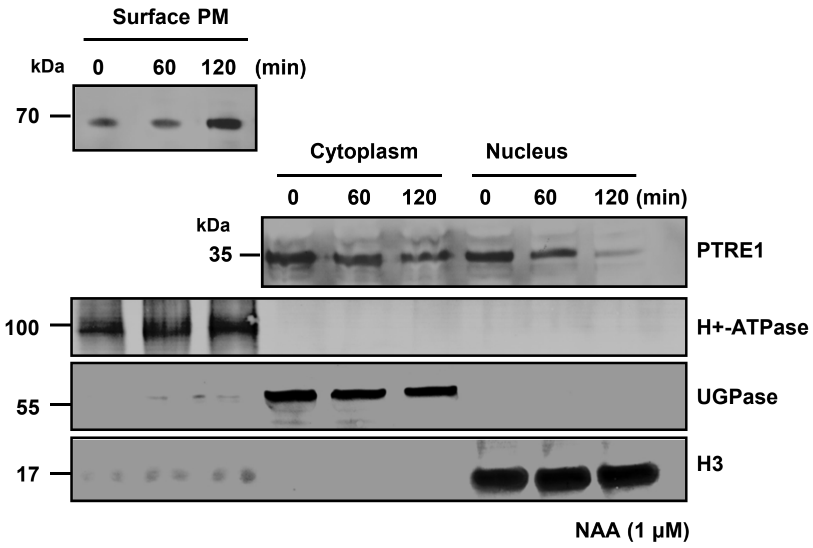

Full uncropped images of replicate blots of Fig. 6a

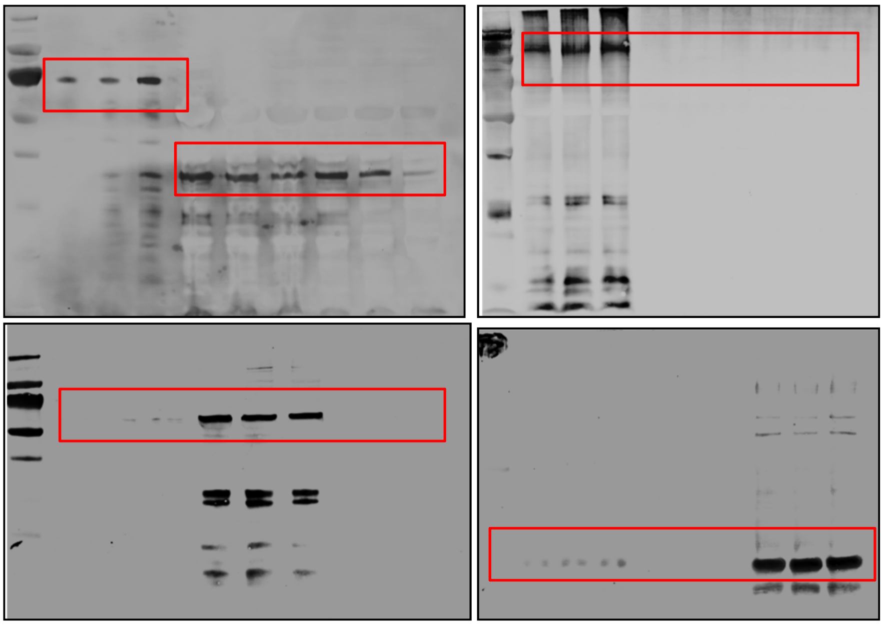

Supplementary Figure 14. Replicate Western blotting analysis of results shown in Fig. 6a. The full uncropped images were shown and cropped bands are indicated by red frames.

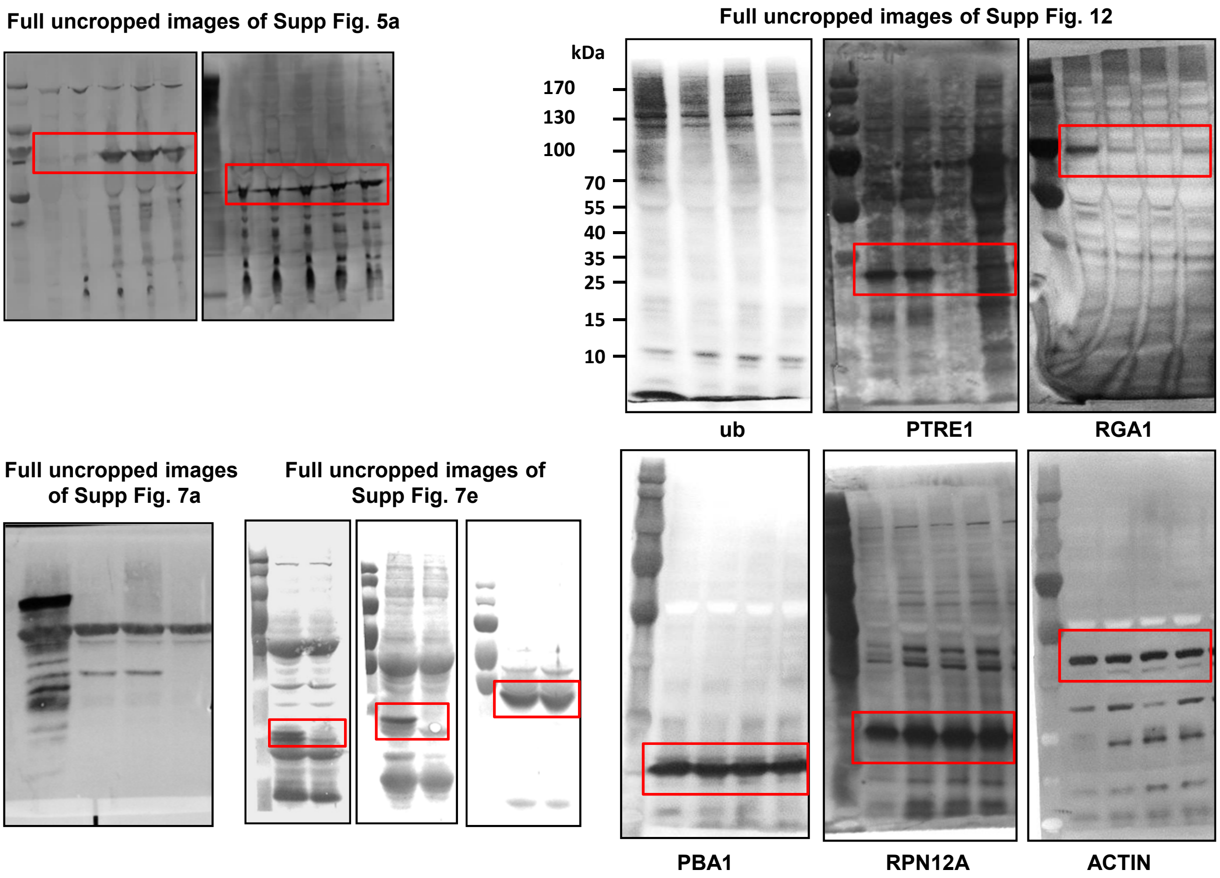

Supplementary Figure 15. Full uncropped images of all gels and blots. The cropped bands shown in supplementary figures 5a, 7a, 7e and 12 are indicated by red frames.

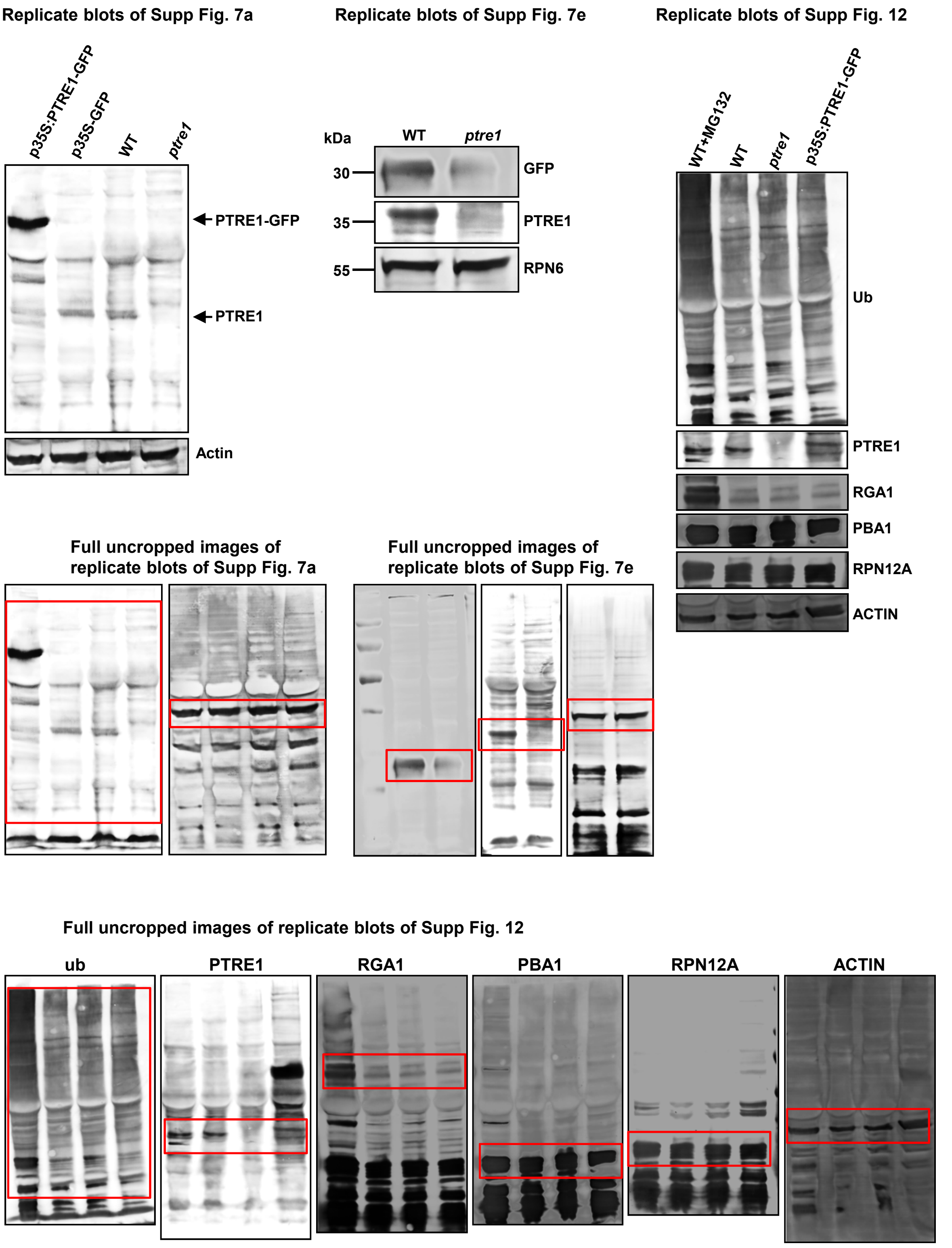

Supplementary Figure 16. Replicate Western blotting analysis of results shown in Supplementary Figs. 7a, 7e and 12. The full uncropped images were shown and cropped bands are indicated by red frames.

Supplemental Table S1. Primers used in this study. Added restriction sites were underlined.

Primer name	Sequence (5'-3')	Added site	Vector	Description
PTRE1-5'	CATGCCATGGCGAATTCTCAGACGG TGA	***Nco* I**	pCAMBIA130 2	PTRE1-GFP fusion; RNA accumulation; identification of *ptre1* mutant
PTRE1-3'	GGACTAGTTATAAAATCTGAACCGC CG	***Spe* I**		
PTRE1-p5'	GGAATTCGTTTACAAAACCAAGTGAA GTCC	***Eco*R I**	pCAMBIA130 0+pBI101.1	pPTRE1:GUS construct
PTRE1-p3'	CGGGATCCCGATTCCGTCTCGAGAG TGTT	***Bam*H I**		
PTRE1-GATE-1	CACCATGGCGAATTCTCAGACG		pENTR/D-TOP O and pGWB5, pGWB654, pGWB14	p35S:PTRE1-G FP/RFP/HA construct
PTRE1-GATE-2	TATAAAATCTGAACCGCC			
UBI10-P1	CCCAAGCTTCGACGAGTCAGTAATA AACG	***Hind* III**	pA7	pUBI10:GUS construct
UBI10-P2	CCGCTCGAGCTGTTAATCAGAAAAA CTCA	***Xho* I**		
pA7-GUS-P1	ACGCGTCGACATGTTACGTCCTGTA GAA	***Sal* I**	pA7	pA7:GUS construct
pA7-GUS-P2	GCTCTAGATCATTGTTTGCCTCCCTG CTG	***Xba* I**		
pA7-LUC-P1	GACTAGTGATGGAAGACGCCAAAA ACATA	***Spe* I**	pA7	pA7:LUC construct
pA7-LUC-P2	CGGGATCCTTACAATTTGGACTTTCC GCC	***Bam*H I**		
pA7-IAA7-P1	ACGCGTCGACATGATCGGCCAACTTA TGAA	***Sal* I**	pA7-LUC	p35S:IAA7-LU C construct
pA7-IAA7-P2	GACTAGTCCAGATCTGTTCTTGCAGT ACTT	***Spe* I**		
pA7-IAA17-P1	ACGCGTCGACATGATGGGCAGTGTCG AGCT	***Sal* I**	pA7-LUC	p35S:IAA17-L UC construct
pA7-IAA17-P2	GACTAGTCCAGCTCTGCTCTTGCAC TTCTC	***Spe* I**		
pA7-IAA19-P1	ACGCGTCGACATGGAGAAGGAAGGA CTCGG	***Sal* I**	pA7-LUC	p35S:IAA19-L UC construct
pA7-IAA19-P2	GACTAGTCCCTCGTCTACTCCTCTAG GCTG	***Spe* I**		
PTRE1-28a5'	CATGCCATGGCGAATTCTCAGACGG	***Nco* I**	pET-28a	PTRE1-His fusion protein
PTRE1-28a3'	CCGGTCGACTATAAAATCTGAACCG CCGG	***Sal* I**		
LB	TGGTTCACGTAGTGGGCCATCG			Genotype analysis of *ptre1* mutant
RP	AACGTAGGCCCAAATTTGATC			
LP	CTCCACAAAACGAAGTTCCAC			
Actin2-1	TCTTCTTCCGCTCTTTCTTTCC			qRT-PCR
Actin2-2	TCTTACAATTTCCCGCTCTGC			
